# Transferrin plays a central role in coagulation balance by interacting with clotting factors

**DOI:** 10.1038/s41422-019-0260-6

**Published:** 2019-12-06

**Authors:** Xiaopeng Tang, Zhiye Zhang, Mingqian Fang, Yajun Han, Gan Wang, Sheng Wang, Min Xue, Yaxiong Li, Li Zhang, Jian Wu, Biqing Yang, James Mwangi, Qiumin Lu, Xiaoping Du, Ren Lai

**Affiliations:** 1 Key Laboratory of Animal Models and Human Disease Mechanisms of Chinese Academy of Sciences/Key Laboratory of Bioactive Peptides of Yunnan Province, Kunming Institute of Zoology, 650223 Kunming, Yunnan China; 2 Kunming College of Life Science, University of Chinese Academy of Sciences, 650204 Kunming, Yunnan China; 3 Key Laboratory of Molecular Biophysics, Huazhong University of Science and Technology, Ministry of Education, College of Life Science and Technology, 430070 Wuhan, Hubei China; 4 Department of Cardiovascular Surgery, Yan’an Affiliated Hospital of Kunming Medical University, 650041 Kunming, Yunnan China; 5 Department of Laboratory, Dehong People’s Hospital, 678400 Dehong, Yunnan China; 6 Department of Pharmacology, University of Illinois at Chicago, Chicago, IL 60612 USA; 7 Institute for Drug Discovery and Development, Chinese Academy of Sciences, 201203 Shanghai, China; 8 KIZ-CUHK Joint Laboratory of Bioresources and Molecular Research in Common Diseases, Kunming Institute of Zoology, Chinese Academy of Sciences, 650223 Kunming, Yunnan China; 9 Sino-African Joint Research Center, Kunming Institute of Zoology, Chinese Academy of Sciences, 650223 Kunming, Yunnan China; 10 Center for Biosafety Mega-Science, Chinese Academy of Sciences, 430071 Wuhan, Hubei China

**Keywords:** Protein-protein interaction networks, Single-molecule biophysics

## Abstract

Coagulation balance is maintained through fine-tuned interactions among clotting factors, whose physiological concentrations vary substantially. In particular, the concentrations of coagulation proteases (pM to nM) are much lower than their natural inactivator antithrombin (AT, ~ 3 μM), suggesting the existence of other coordinators. In the current study, we found that transferrin (normal plasma concentration ~40 μM) interacts with fibrinogen, thrombin, factor XIIa (FXIIa), and AT with different affinity to maintain coagulation balance. Normally, transferrin is sequestered by binding with fibrinogen (normal plasma concentration ~10 μM) at a molar ratio of 4:1. In atherosclerosis, abnormally up-regulated transferrin interacts with and potentiates thrombin/FXIIa and blocks AT’s inactivation effect on coagulation proteases by binding to AT, thus inducing hypercoagulability. In the mouse model, transferrin overexpression aggravated atherosclerosis, whereas transferrin inhibition via shRNA knockdown or treatment with anti-transferrin antibody or designed peptides interfering with transferrin-thrombin/FXIIa interactions alleviated atherosclerosis. Collectively, these findings identify that transferrin is an important clotting regulator and an adjuster in the maintenance of coagulation balance and modifies the coagulation cascade.

## Introduction

Atherosclerosis (AS) is recognized as a chronic inflammatory disease in which plaque accumulates along arteries, leading to serious cardiovascular problems, including heart attack and ischemic stroke.^1,2^ Recent studies have suggested a pro-coagulant state in early AS and an association of AS with venous thrombosis,^3,4^ but the underlying mechanisms remain elusive. A close relationship between blood coagulation and AS has also been revealed by the presence of specific coagulation components in atherosclerotic lesions.^5,6^ Almost all coagulation proteins, including factor XII, factor XI, factor IX, and prothrombin, have been found inside atherosclerotic lesions.^7^ Among coagulation factors, thrombin is a multifunctional serine protease and key enzyme that regulates procoagulant–anticoagulant balance. While fibrin is detectable in atherosclerotic lesions,^8^ direct evidence on the role of thrombin in atherogenic processes comes from the *apolipoprotein E*-deficient (*Apoe*^*−/−*^) mice, a well-established AS model, showing attenuation of atherosclerotic plaque formation upon thrombin inhibition.^9,10^ Thrombin generation can be mediated via the tissue factor–factor VIIa-dependent extrinsic pathway, and can also be triggered through the activation of factor XII^11^ (intrinsic pathway). Factor XII is a coagulation protein that is essential for surface-activated blood coagulation and its deficiency also confers susceptibility to thrombosis according to previous report.^12^ However, factor XII is localized in atherosclerotic plaques and demonstrates significantly higher activity in early rather than advanced atherosclerotic lesions.^3^ Together, these studies reflect the fact that coagulation balance is interrupted in AS.

Nineteen clotting factors have been identified to participate in the coagulation cascade and to maintain thrombohemorrhagic balance.^13–16^ They include the fibrinogen family (fibrinogen (factor I), factor V, factor VIII, and factor XIII),^17^ vitamin K-dependent family ((factor II, thrombin), factor VII, factor IX and factor X),^18,19^ contact family (factor XI, factor XII, high molecular weight kininogen (HMWK), and prekallikrein (PK)),^20,21^ tissue factor (factor III), calcium, Von Willebrand factor (vWf), antithrombin (AT), heparin cofactor-II, protein C, and protein S.^13,20,22–24^ They resemble a coagulation cascade comprised of the intrinsic and the extrinsic pathways, which involve the conversion of each proenzyme into active enzyme by upstream activated clotting factors,^25^ which converge in factor X activation, leading to fibrin activation.^22,26,27^ The normal coagulation pathway represents a balance between the pro-coagulant and the anti-coagulant pathways via fine-tuned interactions among clotting factors.^13,14,28–31^ Normally, the coagulation process is under the control of several inhibitors, i.e., AT, which create a physiological balance between the pro-coagulant and the anti-coagulant pathways to avoid uncontrolled clotting.^13,28,32–35^

Notably, the concentrations of clotting factors vary markedly.^13^ Normal concentrations of fibrinogen, prothrombin, and AT are approximately 10, 2, and 3 μM, respectively, whereas most coagulation proteases fall within the pM to nM range.^14^ As the primary natural inactivator of coagulation proteases, AT limits clot formation, and thus helps avoid thrombus propagation.^14^ Given the huge differences in the concentrations of clotting factors, especially coagulation proteases, which are found at much lower concentrations than their physiological inactivator (AT), we supposed that there exist certain balancers that sequester interactive proteins to orchestrate imbalanced clotting factors in normal plasma. In addition, AS-associated hypercoagulability suggested a dysregulated coagulation. In this study, we found that transferrin, an endogenous plasma protein with high concentration of 40 μM that transports iron,^36,37^ acts as a clotting regulator and an adjuster of coagulation balance by interacting not only with high concentration clotting factors, i.e., fibrinogen and AT, but also with low concentration clotting factors, i.e., coagulation proteases. In AS, up-regulated transferrin interacts with and potentiates thrombin/factor XIIa (FXIIa) and blocks AT’s inactivation effect on coagulation proteases by binding to AT, and thus inducing hypercoagulability.

## Results

### Enhanced enzymatic activity of thrombin and FXIIa is associated with elevated transferrin in AS

**Fig. 1:**
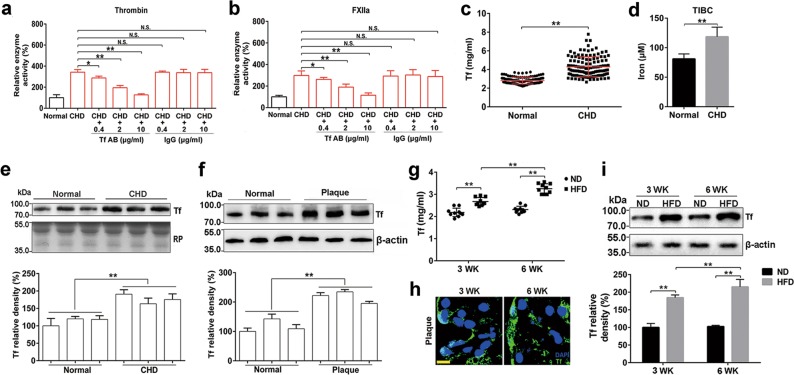
Enhanced enzymatic activity of thrombin and FXIIa is associated with elevated transferrin in atherosclerotic plasma. **a**, **b** An anti-transferrin antibody (Tf AB) alleviated the potentiating ability of CHD plasma on enzymatic activity of thrombin (**a**) and FXIIa (**b**). Data represent mean ± SD (*n* = 6), **P < 0.01 by one-way ANOVA with Dunnett’s post hoc test. **c** Amounts of transferrin in plasma from CHD patients and healthy volunteers were determined by ELISA. Data represent mean ± SD (*n* = 120), **P < 0.01 by unpaired *t*-test. **d** Total iron-binding capacity (TIBC) in plasma from CHD patients and healthy volunteers were determined using TIBC kit. Data represent mean ± SD (*n* = 120), **P < 0.01 by unpaired t-test. **e** Western blot (top) and quantification (bottom) analysis of transferrin in plasma samples from CHD patients and healthy volunteers. Red Ponceau (RP)-stained blots were used as a loading control. **f** Western blot (top) and quantification (bottom) analysis of protein extracts from normal arteries (Normal) and atherosclerotic lesions (Plaque). β-actin was used as a control. Data represent mean ± SD (*n* = 12), **P < 0.01 by unpaired *t*-test (**e**, **f**). **g** Amounts of transferrin in the plasma from the Apoe-/- mice fed with high fat diet (HFD) and normal diet (ND) were determined by ELISA. Data represent mean ± SD (*n* = 10), **P < 0.01 by unpaired *t*-test. **h** Immunofluorescence staining of transferrin (green) in mice atherosclerotic plaque. Cell nuclei were labeled by DAPI. Scale bar represents 10 μm. Images are representative of at least three independent experiments. **i** Western blot (top) and quantification (bottom) analysis of transferrin in aortic roots of the *Apoe*^*−*/*−*^ mice. Data represent mean ± SD (*n* = 10), **P < 0.01 by unpaired *t*-test. N.S.: no significance; Tf: transferrin

To investigate the mechanism responsible for AS-associated hypercoagulability, the enzymatic activities of several coagulation factors, including FVIIa, FXIa, FXIIa, kallikrein, and thrombin, were compared between plasma from healthy volunteers and atherosclerotic patients who have coronary heart disease (CHD) and display angiographically visible luminal narrowing (Supplementary information, Table S1). The enzymatic activities of thrombin and FXIIa in atherosclerotic plasma were 2–2.5 times higher than that in healthy plasma (Supplementary information, Fig. S1a), although total levels of prothrombin and FXII did not differ (Supplementary information, Fig. S1b–d), suggesting the presence of factors potentiating the activation or activities of thrombin/FXIIa in atherosclerotic plasma. The enzymatic activities of kallikrein, FXIa, and FVIIa in plasma did not differ significantly between plasmas from AS patients and normal controls (Supplementary information, Fig. S1a). The factor exhibiting potentiating effects on thrombin/FXIIa was purified and identified as transferrin (Supplementary information, Fig. S2). An anti-transferrin antibody (Tf AB) blocked the elevation of enzymatic activities of thrombin (Fig. 1a) and FXIIa (Fig. 1b) in the atherosclerotic plasma, further suggesting that transferrin is a potentiator for these two coagulation factors. Comparative analysis using enzyme linked immunosorbent assay (ELISA) confirmed that there are elevated transferrin in atherosclerotic plasma (Fig. 1c). The average transferrin concentration in the plasma of CHD patients (*n* = 120, male 60; female 60) was 4.286 mg/mL (SD 1.07) and 4.269 mg/mL (SD 1.03) or 4.303 mg/mL (SD 1.15) in female or male patients, respectively, whereas in healthy individuals (*n* = 120, male 60; female 60) the average concentration was 2.812 mg/mL (SD 0.37) and 2.786 mg/mL (SD 0.29) or 2.837 mg/mL (SD 0.37) in female or male healthy individuals, respectively (Fig. 1c). As illustrated in Fig. 1d, elevated total iron-binding capacity (TIBC) in the plasma of patients mentioned above was consistent with increased transferrin level tested by ELISA (Fig. 1c). Western blot also confirmed that there was elevated transferrin level in atherosclerotic plasma (Fig. 1e). High levels of transferrin were also observed in the atherosclerotic lesion specimens (Fig. 1f; Supplementary information, Table S2).

To further investigate the association of elevated transferrin with AS, *Apoe*^*−/−*^ mice were fed a normal (ND) or a high fat diet (HFD, 21% fat, 0.15% cholesterol) for 6 weeks to test the changes in transferrin in the plasma and atherosclerotic plaque. Notably, elevated transferrin level was observed in the plasma of the HFD-fed *Apoe*^*−/−*^ mice (Fig. 1g), which was congruent with atherosclerotic plaque development (Supplementary information, Fig. S3a). Confocal microscopy and immunoblot analysis also showed increased transferrin in the atherosclerotic plaque (Fig. 1h, i). In addition, quantitative real-time polymerase chain reaction (qRT-PCR) showed that transferrin RNA was dominantly up-regulated in the liver, indicating this organ as the main site of transferrin synthesis (Supplementary information, Fig. S3b).

### Transferrin potentiates thrombin/FXIIa and inhibits AT independently of iron

**Fig. 2:**
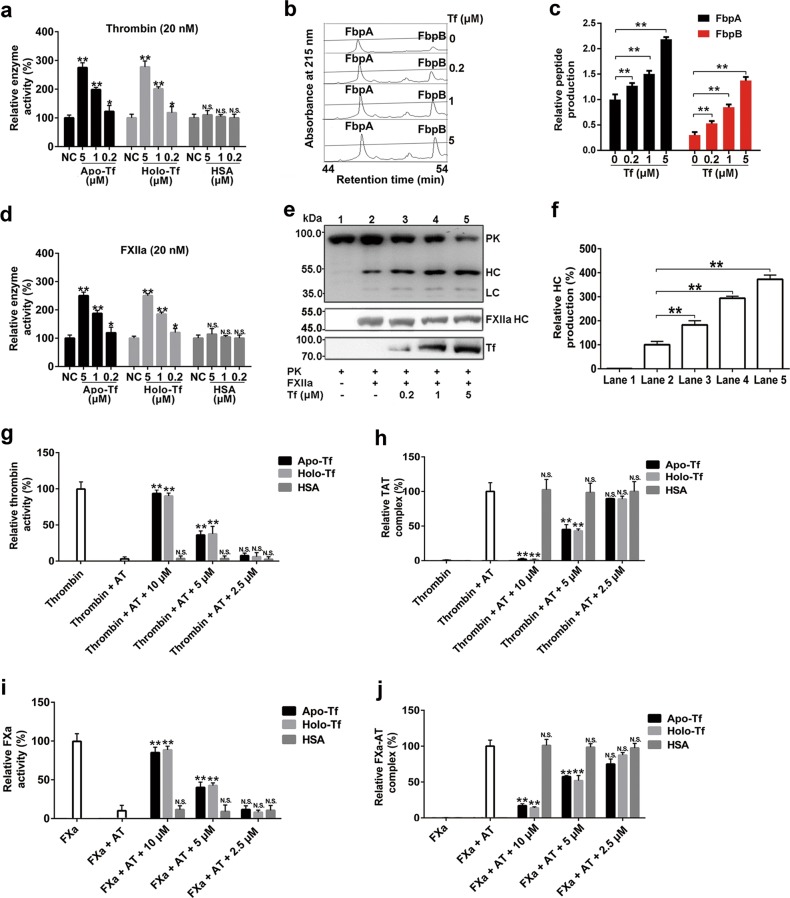
Effects of both apo- and holo-transferrin on thrombin, FXIIa and antithrombin. **a** Potentiating effects of both apo- and holo-transferrin on thrombin. **b**, **c** Representative RP-HPLC analysis (**b**) and quantification (**c**) of fibrinopeptide A (FbpA) and fibrinopeptide B (FbpB) released from 5 mg of fibrinogen hydrolyzed by 0.1 NIH unit thrombin mixed with 0, 0.2, 1, or 5 μM apo-transferrin, respectively. **d** Potentiating effects of both apo- and holo-transferrin on FXIIa. **e**, **f** Representative western blot (**e**) and quantification analysis of kallikrein heavy chain (HC ∼52 kDa) (**f**) released from 10 μg of prekallikrein (PK) hydrolyzed by 0.01 NIH unit FXIIa mixed with 0, 0.2, 1, or 5 μM apo-transferrin (lane 2–5), respectively. Blots of PK, FXIIa heavy chain (FXIIa HC), transferrin, and kallikrein light chain (LC ∼36 and 33 kDa) are also shown. **g**–**j** Apo- and holo-transferrin block antithrombin (AT)’s inactivation effect on thrombin (**g**, **h**) and FXa (**i**, **j**). TAT: thrombin–AT complex. HSA: human serum albumin. Data represent mean ± SD of five independent experiments, *P < 0.05, **P < 0.01 by one-way ANOVA with Dunnett’s post hoc test. N.S.: no significance; Tf: transferrin

As an iron carrier, transferrin exists in plasma in both the ferric iron-bound state (holo-transferrin) and unbound state (apo-transferrin). As illustrated in Fig. 2a, d, both apo- and holo-transferrin were found to show a similar effect to enhance the enzymatic activities of thrombin and FXIIa. At the concentrations of 0.2, 1 and 5 μM, transferrin enhanced the enzymatic activity of thrombin by ∼0.2-, 1- and 1.8-fold, and that of FXIIa by ∼0.2-, 0.7- and 1.5-fold, respectively. Similarly, apo- and holo-transferrin exhibited no differences in their promotion of coagulation by shortening the recalcification time (Supplementary information, Fig. S4). Transferrin also enhanced the ability of thrombin and FXIIa to hydrolyze their natural substrates, i.e., fibrinogen (Fig. 2b, c) and prekallikrein (PK) (Fig. 2e, f), respectively. Fibrinopeptide A (FbpA) and FbpB, which result from fibrinogen hydrolysis by thrombin, increased ∼0.2-, 0.5-, and 1.2-fold and ∼1.1-, 2.1-, and 4.2-fold, respectively, after 30 min of treatment with transferrin at 0.2, 1, and 5 μM (Fig. 2b, c). At the concentrations of 0.2, 1, and 5 μM, transferrin also increased the ability of FXIIa to release the hydrolytic product of PK (kallikrein heavy chain (HC), 52 kDa) by ∼0.8-, 1.9- and 2.7-fold, respectively (Fig. 2e, f). Transferrin showed no effects on zymogen activation of thrombin or FXIIa or on the activities of kallikrein, FXIa, or FVIIa (Supplementary information, Fig. S5a–c). As illustrated in Fig. 2g, i, both apo- and holo-transferrin blocked the inhibitory activity of AT toward thrombin and FXa. The inactivation on thrombin and FXa by 2 μM AT was completely blocked by 10 μM transferrin. As a result, the generation of thrombin–AT (TAT) and FXa–AT complexes was blocked (Fig. 2h, j). In addition, thrombin-induced platelet aggregation was augmented by transferrin (Supplementary information, Fig. S6). These data indicate that transferrin can induce hypercoagulability by potentiating thrombin and FXIIa and blocking inactivation effect of AT on thrombin and FXa.

### Transferrin directly interacts with thrombin, FXIIa, fibrinogen, and AT

**Fig. 3:**
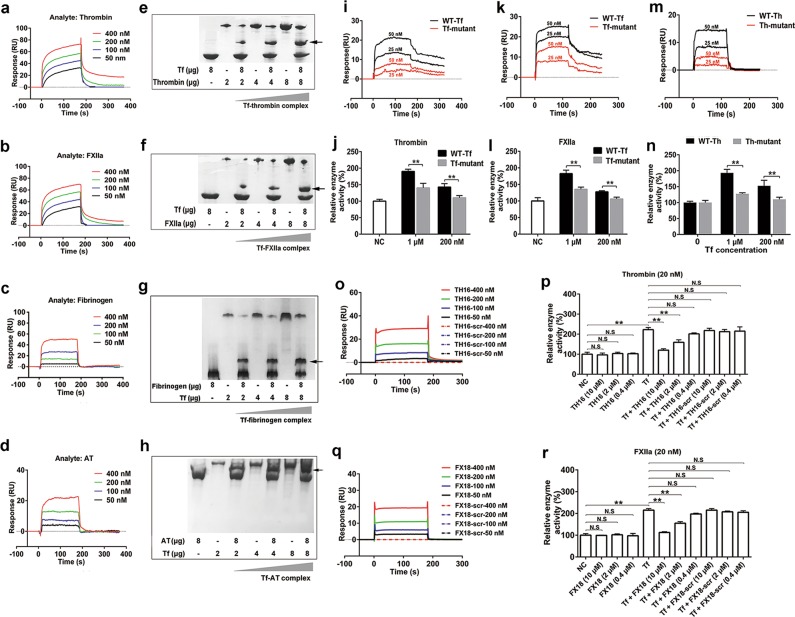
Interactions between transferrin and clotting factors. **a**–**d** SPR analysis of the interaction between transferrin and thrombin (**a**), FXIIa (**b**), fibrinogen (**c**) or antithrombin (AT) (**d**). **e**, **f** Native gel shift analysis of interaction between transferrin (8 μg) and thrombin (2, 4, and 8 μg) (**e**) or FXIIa (2, 4, and 8 μg) (**f**). **g**, **h** Native gel shift analysis of interaction between transferrin (2, 4, and 8 μg) and fibrinogen (8 μg) (**g**) or AT (8 μg) (**h**). Arrows indicate the complexes of transferrin–thrombin, transferrin–FXIIa, transferrin–fibrinogen or transferrin–AT. **i**, **k** SPR analysis of the interaction between wild-type transferrin (WT-Tf) or transferrin mutant (E333,338R) and thrombin (**i**) or FXIIa (**k**). **j**, **l** Effects of wild-type transferrin and transferrin mutant on enzymatic activity of thrombin (**j**) and FXIIa (**l**). **m** SPR analysis of the interaction between transferrin and wild-type thrombin (WT-Th) or thrombin mutant (Th-mutant, R117,122A).** n** Effects of transferrin on enzymatic activity of wild-type thrombin and thrombin mutant. **o**, **q** SPR analysis of interaction between transferrin and TH16 or TH16-scr (scrambled control of TH16) (**o**), and FX18 or FX18-scr (scrambled control of FX18) (**q**). **p** Effects of TH16 and TH16-scr on potentiating activity of transferrin on thrombin. **r** Effects of FX18 and FX18-scr on the potentiating activity of transferrin on FXIIa. Data represent mean ± SD of six independent experiments, **P < 0.01 by unpaired *t*-test (**j**, **l**, **n**). **P **<** 0.01 by one-way ANOVA with Dunnett’s post hoc test (**p**, **r**). Tf: transferrin

Surface plasmon resonance (SPR) analysis revealed that apo-transferrin directly interacted with thrombin (Fig. 3a), FXIIa (Fig. 3b), fibrinogen (Fig. 3c) and AT (Fig. 3d), whereas human serum albumin (HSA, negative control) shows no interaction with them (Supplementary information, Fig. S5d). The binding molar ratios between apo-transferrin and fibrinogen and apo-transferrin and AT were 4:1 and 2:1 (Supplementary information, Fig. S5e). The association rate constant (*Ka*), dissociation rate constant (*Kd*), and equilibrium dissociation constant (*KD*) values for the interaction between apo-transferrin and thrombin were 4.7 × 10^5^ M^−1^ s^−1^, 3.6 × 10^−3^ s^−1^, and 7.7 nM, and those for apo-transferrin–FXIIa interaction were 1.8 × 10^5^ M^−1^ s^−1^, 2.5 × 10^−3^ s^−1^, and 13.9 nM, respectively (Supplementary information, Table S3). Furthermore, the parameter values were 3.4 × 10^4^ M^−1^ s^−1^, 1 × 10^−3^ s^−1^, and 29 nM for apo-transferrin–fibrinogen interaction, and 2.1 × 10^3^ M^−1^ s^−1^, 1.1 × 10^−3^ s^−1^, and 524 nM for apo-transferrin–AT interaction, respectively (Supplementary information, Table S3). The native gel shift assays also showed a complex formation between apo-transferrin and thrombin (Fig. 3e), FXIIa (Fig. 3f), fibrinogen (Fig. 3g), and AT (Fig. 3h). SPR analysis also revealed that apo-transferrin interacted with prothrombin and FXII (Supplementary information, Fig. S5f, g). The *Ka*, *Kd*, and *KD* for apo-transferrin–prothrombin interaction were 1.4 × 10^5^ M^−1^ s^−1^, 2.5 × 10^−3^ s^−1^, and 18 nM, respectively, and for apo-transferrin–FXII interaction were 0.3 × 10^5^ M^−1^ s^−1^, 1.2 × 10^−3^ s^−1^, and 40 nM, respectively. Holo-transferrin also interacted with thrombin, FXIIa, fibrinogen, AT, prothrombin, and FXII with the similar properties as observed for apo-transferrin (Supplementary information, Fig. S5h).

We next determined the actual protein–protein binding properties behind the complex formations between transferrin and thrombin/FXIIa. Docking model of the transferrin–thrombin complex indicated that the conformation of thrombin exosite I was affected by the binding of transferrin via several electrostatic interactions and one pool hydrophobic effect (Supplementary information, Fig. S7a). The binding of transferrin opens and widens the groove-like exosite I site. This conformation change results in a slight shift in the catalytic triad, which is located adjacent to exosite I. Therefore, substrates of thrombin have more space to access the triad residues. Similar to the transferrin–thrombin complex, FXIIa interacts with transferrin through its own groove-like domain (exosite I domain analogue), which exhibits almost the same folding mode and changes in conformation as the exosite I of thrombin (Supplementary information, Fig. S7b).

Based on the docking model and the structural characteristics of the transferrin–thrombin/FXIIa complex, we found two key residues of transferrin (E333 and E338) that may play key roles in the interaction of transferrin with thrombin/FXIIa. The mutant of transferrin (E333,338R) was thus constructed (Supplementary information, Fig. S8a–e). Notably, the transferrin mutant exhibited weak interaction with thrombin (Fig. 3i) and FXIIa (Fig. 3k). The ability of the transferrin mutant to potentiate thrombin (Fig. 3j) and FXIIa (Fig. 3l) was significantly decreased compared with the wild-type transferrin. Furthermore, the docking model also indicated that two key residues of thrombin (R3 and R122) play key roles in the interaction of thrombin with transferrin. The corresponding mutant of thrombin (R117,122A) (Supplementary information, Fig. S8f–h) exhibited significantly weaker interaction with transferrin compared with wild-type thrombin (Fig. 3m). Although the enzymatic activity of the thrombin mutant was not influenced, the ability of transferrin to potentiate the thrombin mutant was significantly decreased (Fig. 3n).

Given that transferrin potentiates thrombin and FXIIa by interacting with them, we hypothesized that specific peptides at the binding sites of thrombin and FXIIa may competitively bind to the same target in transferrin and thus inhibit its effects on thrombin and FXIIa. Exosite I motif-based inhibitor peptides TH16 (RIGKHSRTRYERNIEK) and FX18 (RRNHSCEPCQTLAVRSYR) were designed. SPR analysis revealed critical interaction between transferrin and TH16 (Fig. 3o) or FX18 (Fig. 3q). The *Ka*, *Kd*, and *KD* values for transferrin–TH16 were 3.1 × 10^3^ M^−1^ s^−1^, 0.3 × 10^−3^ s^−1^, and 97 nM, and for transferrin –FX18 were 2.8 × 10^3^ M^−1^ s^−1^, 0.4 × 10^−3^ s^−1^, and 143 nM, respectively. However, the scrambled peptides of TH16 (TH16-scr, RKKGIRRYTERHSNIE) and FX18 (FX18-scr, SCPTHYSRQRCRNAVLER) showed no interaction with transferrin. Functional study demonstrated that both TH16 and FX18 inhibited the potentiating activity of transferrin on thrombin (Fig. 3p) and FXIIa (Fig. 3r) in a dose-dependent manner. TH16 or FX18 alone showed no effect on the enzymatic activity of thrombin or FXIIa even at concentrations of 10 μM.

We next performed in silico studies using 20 nanosecond molecular dynamics (MD) simulations to evaluate the stability of the AT–transferrin complex. Result showed that transferrin can steadily bind around the reactive center loop (RCL) of AT (Supplementary information, Fig. S7c), indicating that transferrin suppressed the inhibitory effects of AT by interfering with the interaction of AT and other coagulation factors, such as thrombin or FXa, etc. Furthermore, SPR results verified that transferrin could bind with the synthetic peptide (RCL19: EAAASTAV VIAGRSLNPNR) which is from the RCL of AT (Supplementary information, Fig. S7d), and further verified transferrin’s interaction with the RCL of AT. However, the scrambled peptide of RCL19 (RCL19-scr, GANPRELNSAATIASVARV) showed no interaction with transferrin (Supplementary information, Fig. S7d). The *Ka*, *Kd*, and *KD* values for transferrin–RCL19 were 1.8 × 10^3^ M^−1^ s^−1^, 1.0 × 10^−3^ s^−1^, and 556 nM.

### Increased transferrin–prothrombin/thrombin and transferrin–FXII/FXIIa complexes in human atherosclerotic plasma and lesions

**Fig. 4:**
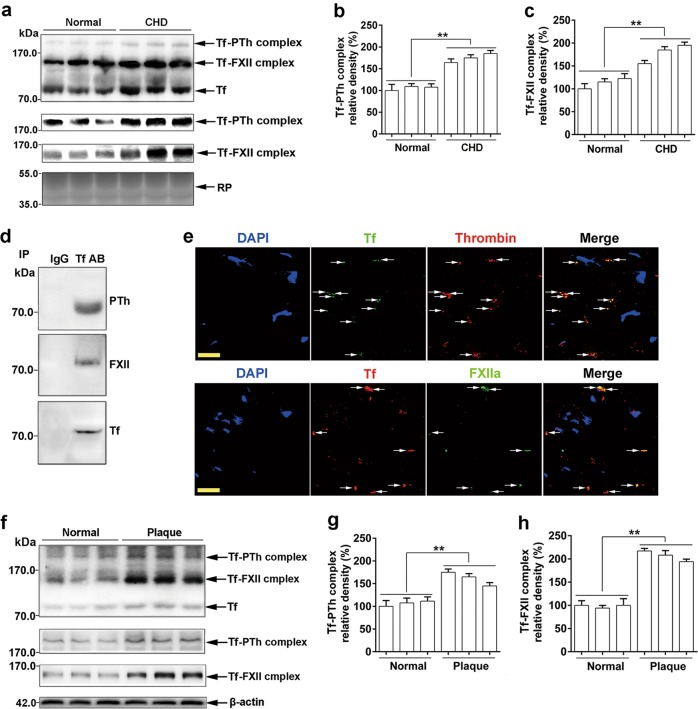
Elevated levels of transferrin–thrombin/FXIIa complexes in CHD patient plasma and atherosclerotic plaque. **a** Western blot analysis of transferrin–prothrombin (Tf–PTh) and transferrin–FXII complexes in healthy (Normal) and CHD plasma. Red Ponceau (RP)-stained blots were uesd as the loading control. **b**, **c** Quantification of the transferrin–PTh (**b**) and transferrin–FXII (**c**) complexes. **d** Co-immunoprecipitation of transferrin and prothrombin or FXII in human normal plasma. **e** Human atherosclerotic plaque was labeled with either anti- transferrin antibody (green) or anti-thrombin antibody (red) to detect presence of the transferrin–thrombin complex (top), or labeled with either anti-transferrin antibody (red) or anti-FXIIa antibody (green) to detect presence of the transferrin–FXIIa complex (bottom). Cell nuclei were labeled with DAPI. Arrows indicate transferrin–thrombin- or transferrin–FXIIa-positive structures. Scale bar represents 30 μm. Images are representative of at least three independent experiments. **f**–**h** Western blot analysis (**f**) and quantification of transferrin–prothrombin complex (**g**) and the transferrin–FXII complex (**h**) in the supernatants of the homogenized thoracic aorta tissue from normal controls and atherosclerotic patients. Data represent mean ± SD (*n* = 12), **P < 0.01 by unpaired *t*-test. Tf: transferrin

To confirm whether transferrin forms complexes with thrombin/FXIIa in atherosclerotic plasma or lesions, we used Bis (sulfosuccinimidyl) suberate (BS^3^) to stabilize the possible components that complex with transferrin. Western blot analysis using anti-transferrin antibody showed three bands (Fig. 4a, the top panel) including transferrin, and transferrin–prothrombin/FXII complexs confirmed by antibodies against prothrombin and FXII (Fig. 4a), respectively. The amount of the transferrin–prothrombin/FXII complexes in the plasma of CHD patients was higher than that in normal controls (Fig. 4b, c). Co-immunoprecipitation analysis further revealed the formation of transferrin–prothrombin and transferrin–FXII complexes in plasma (Fig. 4d). The presence of transferrin- and thrombin-/FXIIa-positive deposits indicated the formation of transferrin–thrombin/FXIIa complexes in atherosclerotic plaque (Fig. 4e). Moreover, the formation of transferrin–prothrombin and transferrin–FXII complexes was also observed in atherosclerotic plaque by western blot analysis after cross-linking, with the amount found to be greater than that in normal controls (Fig. 4f–h).

### Transferrin overexpression aggravates atherosclerotic lesions and hypercoagulability, which are attenuated by transferrin down-regulation

**Fig. 5:**
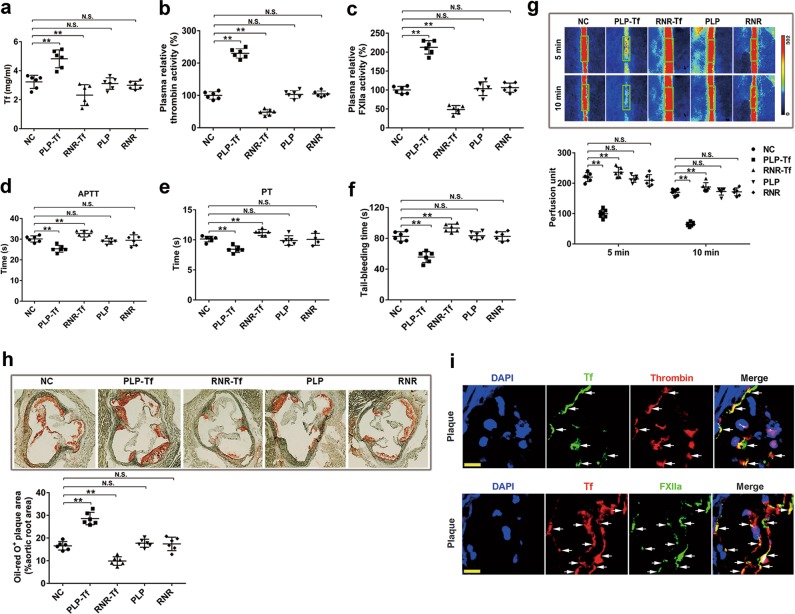
Effects of transferrin overexpression and knockdown on atherosclerotic development and hypercoagulability. **a–f** Plasma concentrations of transferrin in five groups of *Apoe*^*−/−*^ mice fed a HFD for 6 weeks (transferrin overexpression (PLP-Tf) and its blank PLP, knockdown (RNR-Tf) and its blank RNR, and normal *Apoe*^*−/−*^ mice (NC)) (**a**). Relative activity of thrombin (**b**) and FXIIa (**c**), APTT (**d**), PT (**e**) in their plasma and tail bleeding time (**f**) are also shown. **g** Representative images of carotid artery blood flow (top) in FeCl_3_-treated mice by laser speckle perfusion imaging, and the region of interest (green rectangle) was placed in the carotid artery to quantify blood flow change. Relative blood flow in the region of interest is shown (bottom) by using perfusion unit. Red: blood flow; Blue and black area: background; The color bar on the right side indicates the perfusion unit scale (0–302). **h** Representative images of oil-red O-stained atherosclerotic plaques (top) and quantitative analysis of stained area (bottom) are shown. Data represent mean ± SD (*n* = 6), **P < 0.01 by one-way ANOVA with Dunnett’s post hoc test. **i** Atherosclerotic plaques from the mice fed a HFD for 4 weeks were labeled with either anti-transferrin, anti-thrombin, or anti-FXIIa antibodies. Cell nuclei were labeled by DAPI. Arrows indicate transferrin–thrombin- or transferrin–FXIIa-positive structures. Scale bar represents 30 μm. Images are representative of at least three independent experiments. N.S.: no significance; Tf: transferrin

To elucidate the role of transferrin in atherosclerotic lesion development, the effects of transferrin overexpression and knockdown on the development of AS and hypercoagulability were evaluated. Transferrin expression levels were first validated by qRT-PCR and western blot (Supplementary information, Fig. S9). After virus injection, *Apoe*^*−/−*^ mice were fed a HFD for 6 weeks to study the development of AS and hypercoagulability. As illustrated in Fig. 5a, an increase in transferrin plasma concentration was observed in transferrin-overexpressed *Apoe*^*−/−*^ mice (PLP-Tf) compared with *Apoe*^*−/−*^ controls (NC) or blank virus (with empty overexpression (PLP) vector)-injected mice. Conversely, a reduction in transferrin plasma concentration was found in transferrin-knockdown *Apoe*^*−/−*^ mice (RNR-Tf) in comparison with *Apoe*^*−/−*^ controls or blank virus (with empty knockdown (RNR) vector)-injected mice. The plasma from PLP-Tf mice showed significantly elevated activities of thrombin (Fig. 5b) and FXIIa (Fig. 5c) as analyzed using synthetic substrates, and displayed shortened activated partial thromboplastin time (APTT) (Fig. 5d), prothrombin time (PT) (Fig. 5e), and tail bleeding time (Fig. 5f). In contrast, the plasma from RNR-Tf mice showed significantly reduced thrombin/FXIIa activities and prolonged APTT, PT, and tail bleeding time (Fig. 5b–f), suggesting that plasma transferrin regulates enzymatic activities of thrombin and FXIIa as well as APTT, PT, and tail bleeding time. No significant changes in the iron metabolism and erythrocyte indices were observed after transferrin overexpression or knockdown (Supplementary information, Table S4).

The role of transferrin in promoting hypercoagulability was further investigated in a mouse thrombosis model induced by FeCl_3_. As illustrated in Fig. 5g, blood flow in the carotid artery was significantly decreased in transferrin-overexpressed mice, suggesting an increased thrombus formation. Inversely, blood flow was increased in the transferrin-knockdown mice, suggesting inhibited thrombosis. Consequently, as illustrated in Fig. 5h, a substantial increase in plaque size in the aortic root was observed in transferrin-overexpressed *Apoe*^*−/−*^ mice, whereas reduced lesion formation was found in transferrin-knockdown *Apoe*^*−/−*^ mice. In addition, the transferrin- and thrombin/FXIIa-positive deposits (Fig. 5i) were also observed in the atherosclerotic lesions of *Apoe*^*−/−*^ mice. These findings demonstrate that transferrin participates in maintaining coagulation balance and pathogenesis of AS.

### Interference with transferrin exerts anti-hypercoagulability and anti-AS effects

**Fig. 6:**
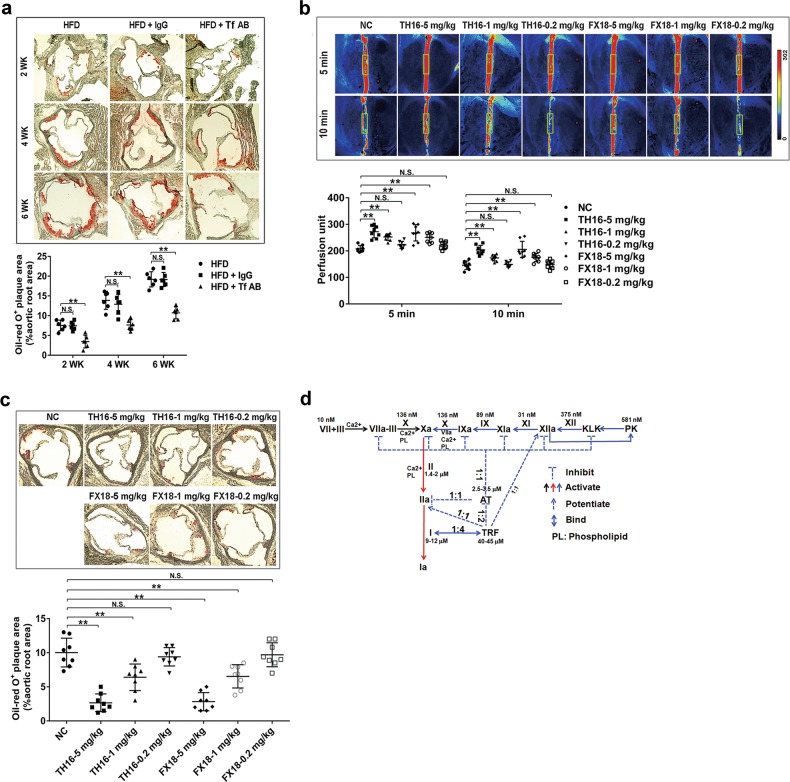
Transferrin interferences exert anti-AS effects in vivo. The HFD-fed *Apoe*^*−/−*^ mice were subjected to anti-transferrin antibody (Tf AB) or control IgG treatment twice/week for 6 weeks. **a** Representative images (top) of oil-red O-stained plaques and quantitative analysis (bottom) of the stained area are shown. **b** Effects of TH16 and FX18 on FeCl_3_-induced carotid artery thrombus formation in C57BL/6J mice. Representative images of carotid artery blood flow (top) and quantitation (bottom) are shown. Red: blood flow; Blue and black area: background; The color bar on the right side indicates the perfusion unit scale (0–302). **c** Effects of TH16 and FX18 on mouse AS development. Representative images (top) of oil-red O-stained plaques and quantitative analysis (bottom) of the stained area are shown. **d** Graphical representation of transferrin’s central role and its interactions with clotting factors to maintain coagulation balance. Transferrin participates in three types of interactions for coagulation balance including: 1) most of transferrin (TRF, ~40 μM) is sequestered by binding with fibrinogen (~10 μM) at a molar rate of 4:1; 2) transferrin blocks inactivation effect of AT towards thrombin and FXa by binding with AT at a molar rate of 2:1; 3) transferrin interacts and potentiates thrombin and FXIIa at a molar rate of 1:1. Data represent mean ± SD (*n* = 6–8), **P < 0.01 by one-way ANOVA with Dunnett’s post hoc test. N.S.: no significance

After a 6-week treatment with an anti-transferrin antibody, a decrease in transferrin plasma concentration was observed (Supplementary information, Fig. S10a). This was accompanied by a relative decrease in the enzymatic activities of thrombin (Supplementary information, Fig. S10b) and FXIIa (Supplementary information, Fig. S10c), as well as prolonged APTT (Supplementary information, Fig. S10d), PT (Supplementary information, Fig. S10e), and tail bleeding time (Supplementary information, Fig. S10f), indicating decreased hypercoagulability in *Apoe*^*−/−*^ mice with transferrin depletion. Consequently, an obvious decrease in plaque size was observed in the anti-transferrin antibody-treated *Apoe*^*−/−*^ mice (Fig. 6a). Importantly, no statistically significant changes in the iron metabolism and erythrocyte indices were observed after anti-transferrin antibody treatment (Supplementary information, Table S4).

As illustrated in Fig. 6b, increased blood flow in the carotid artery in vivo indicated an inhibited thrombus formation by exosite I motif-based peptides TH16 and FX18, which only inhibited transferrin’s potentiating ability on thrombin and FXIIa without direct effect on the enzymes (Fig. 3p, r). Moreover, after 3 weeks of treatment, TH16 and FX18 significantly decreased atherosclerotic plaque size in a dose-dependent manner, suggesting anti-AS effects (Fig. 6c). Together, these data indicate that transferrin drives the development of atherosclerotic plaque and that interference with transferrin may provide valuable opportunities for AS prevention and treatment.

### Transferrin participates in the regulation of blood coagulation in physiological conditions

**Fig. 7:**
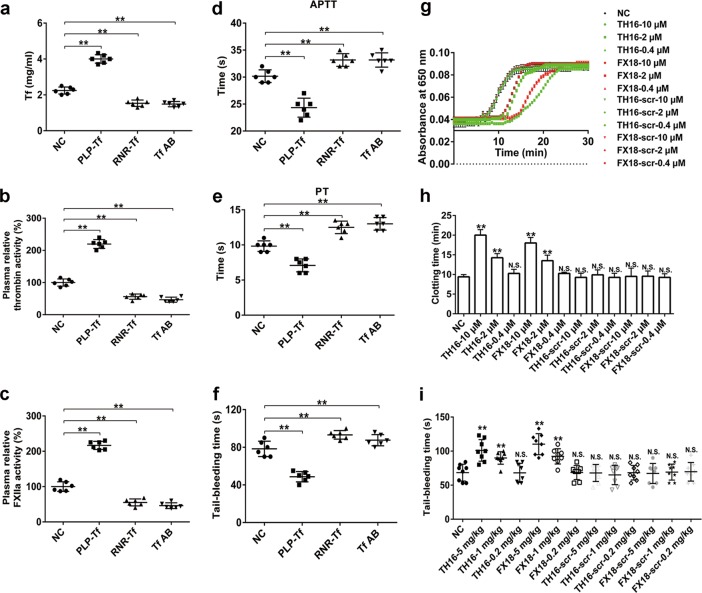
Effects of transferrin overexpression, knockdown, anti-transferrin antibody treatment, and interference peptides on coagulation. **a** Plasma concentrations of transferrin in four groups of C57BL/6J mice (transferrin overexpression (PLP-Tf), knockdown (RNR-Tf), anti-transferrin antibody-treated (Tf AB), and normal control mice (NC)). **b–f** Relative activity of thrombin (**b**) and FXIIa (**c**), APTT (**d**), PT (**e**) in their plasma and tail bleeding time (**f**) are also shown. **g**–**i** Effects of TH16, FX18, TH16-scr, and FX18-scr on plasma recalcification time (**g**), clotting time (**h**), and tail bleeding time (**i**) in C57BL/6J mice. Data represent mean ± SD (*n* = 6–8), **P < 0.01 by one-way ANOVA with Dunnett’s post hoc test. N.S.: no significance; Tf: transferrin

To further investigate the role of transferrin in coagulation, the sequelae of transferrin overexpression, knockdown, anti-transferrin antibody treatment, and interference peptide treatment (TH16 and FX18) in C57BL/6J mice were evaluated (Fig. 7). Enzymatic activities of thrombin and FXIIa were elevated and APTT, PT, and tail bleeding time were shortened upon transferrin overexpression, suggesting a hypercoagulability state. In contrast, the plasma from transferrin-knockdown and anti-transferrin antibody-treated mice showed significantly reduced thrombin and FXIIa activities and prolonged APTT, PT, and tail bleeding time (Fig. 7b–f). In addition, TH16 and FX18 prolonged plasma recalcification time in a dose-dependent manner, suggesting that coagulation was inhibited by the two peptides (Fig. 7g, h). The increase in plasma recalcification time may further contribute to the extended bleeding time in mice (Fig. 7i).

## Discussion

The balance between clotting and bleeding is always maintained by complicated interactions between the coagulation and anti-coagulation systems in the body under normal physiological conditions. The coagulation process is controlled by several inhibitors, which limit clot formation and thus avoid thrombus propagation.^13,14^ There are several important natural inhibitors of coagulation enzymes in plasma like AT, protein C, protein S, and TFPI, and AT is the principal inhibitor that inactivate thrombin, FXa, and FIXa, etc.^13^ However, the concentrations of coagulation proteases (from pM to nM) are much lower than that of AT (~3 μM), suggesting that coagulation protease functions can be largely blocked by AT in normal circulation. Pro-coagulant state is found in CHD plasma, i.e., AS, suggesting that coagulation balance is interrupted and AT’s inactivation effect on coagulation proteases is inhibited; to date, however, whether this process actually occurs remains unknown. In the current study, we found enhanced thrombin and FXIIa enzymatic activity in atherosclerotic plasma, although total levels of prothrombin and FXII did not differ from that in normal plasma (prothrombin ~ 2 μM, FXII ~ 0.375 μM).^38^ Thus the concentrations of thrombin and FXIIa were less than 2 and 0.375 μM, respectively, lower than the concentration of AT (~3 μM). Considering that AT binds and inactivates coagulation enzymes at a ratio of 1:1, we inferred that another plasma factor may block AT to contribute to the enhanced enzymatic activity of thrombin and FXIIa and to induce the pro-coagulant state in AS plasma.

It was proposed in a previous report in the 1980s that iron deficiency would be protective against CHD as a potential explanation for the differences in CHD risk between men and women.^39^ However, two studies have well documented that iron deficiency is associated with an increased risk of cardiovascular disease.^40,41^ In addition, our extended preprinted research also suggests that the upregulated transferrin in iron deficiency is a potential mediator in inducing hypercoagulability and cardiovascular disease.^42^ Furthermore, high iron stores has been suggested as risk factor for CHD.^43^ Iron overload can aggravate AS by inducing oxidative stress and inflammation according to a recent report.^44^ Study has demonstrated that ferritin, the iron storage protein, is highly expressed in human atherosclerotic lesions.^45^ It has been reported that transferrin receptor 1 and ferritin were highly expressed in foamy macrophages and smooth muscle cells in intimal lesions of human carotid atheroma, in association with the severity of plaques.^46^ In addition to ferritin at the intracellular level, circulating iron homeostasis is also regulated by transferrin, which binds ferric ions and transports them to transferrin receptor on cells.^36,37^ To date, whether transferrin is related to coagulation and AS remains unclear. Decreased transferrin level in inflammation and cardiovascular disease has been described in a previous report.^47^ However, some other articles suggested an elevated level of transferrin in cardiovascular disease.^48,49^ Such controversial issue needs to be clarified with substantial evidence. In this study, we found elevated levels of transferrin in human atherosclerotic plasma, which were associated with enhanced enzymatic activity of thrombin and FXIIa and a pro-coagulant state (Fig. 1a–d; Supplementary information, Figs. S1 and S2). The concentration of transferrin in the CHD plasma samples was 53% higher than that in the controls (4.286 vs 2.812 mg/mL). Additionally, elevated transferrin levels were also observed in the plasma and atherosclerotic plaque of AS mice (Fig. 1f–h). Further study indicated that transferrin directly interacted with fibrinogen, thrombin, FXIIa, and AT with different affinities to maintain coagulation balance. The *KD* values of the interactions between transferrin and thrombin, FXIIa, fibrinogen, and AT were 7.7, 13.9, 29, and 524 nM, respectively (Fig. 3a–d). Transferrin showed the ability to potentiate the enzymatic activities of thrombin and FXIIa, two key coagulation proteases. Transferrin can exist in plasma as either holo-transferrin or apo-transferrin, with 30–40% present as holo-transferrin,^50–52^ suggesting surplus transferrin may have other physiological functions. We found that both holo- and apo-transferrin showed similar binding abilities for thrombin, FXIIa, fibrinogen, and AT. They also showed similar activities to potentiate thrombin and FXIIa and to block AT’s inactivation effect on thrombin and FXa (Fig. 2). All these findings provide evidence for the iron-independent function of transferrin in coagulation and further suggest the dual role of transferrin in coagulation and as an iron carrier. The formation of transferrin–prothrombin/thrombin and transferrin–FXII/FXIIa complexes both in vivo and in vitro was further confirmed by SPR, native-PAGE, immunoprecipitation and laser confocal microscopy (Figs. 3a–h and 4). Importantly, transferrin bound with fibrinogen at a molar ratio of 4:1, consistent with their molar concentration ratio in normal plasma (40 and 10 μM) (Supplementary information, Fig. S5e). In addition, transferrin also bound with AT, thrombin and FXIIa at a molar ratio of 2:1, 1:1, and 1:1, respectively (Supplementary information, Fig. S5e). All these suggest that most transferrin proteins were sequestered from coagulation proteases and AT by fibrinogen to maintain coagulation balance in normal plasma, whereas up-regulated transferrin in AS plasma interacted with and potentiated thrombin and FXIIa and blocked the inactivation effect of AT on thrombin and FXa to induce hypercoagulability (Fig. 6d).

Transferrin’s role as a procoagulant factor was proven by high thrombotic and atherosclerotic risks in transferrin-overexpressed mice and alleviated atherosclerotic lesion formation and coagulability in mice following transferrin interference (transferrin knockdown, neutralization with anti-transferrin antibody) (Figs. 5 and  6a; Supplementary information, Fig. S10a–f). The docking model and the structural characteristics of the transferrin–thrombin and transferrin–FXIIa complexes suggest that the binding of transferrin to exosite I on thrombin or the exosite I domain analogue on FXIIa may affect the steric hindrance of the active site cleft. Based on the exosite I domains of thrombin and FXIIa, the inhibitor peptides (TH16 and FX18) were developed. These two peptides inhibited the potentiating activity of transferrin on thrombin and FXIIa in vitro (Fig. 3p, r) and alleviated hypercoagulability and atherosclerotic lesions in vivo (Figs. 6b, c and 7g–i), further confirming that the binding sites of thrombin and FXIIa towards transferrin are located in the exosite I domains, and exosite I motif-based interference of transferrin–thrombin/FXIIa interaction represents a novel strategy for the development of clinical anti-AS treatment.

Collectively, our data have demonstrated that transferrin plays a central role in maintaining coagulation balance via interaction with coagulation and anti-coagulation factors. Here, the old protein transferrin was proven to be a coagulation regulator. Normally, coagulation balance was achieved by the two interactions: 1) transferrin–fibrinogen at a molar ratio of 4:1, and 2) AT-coagulation proteases, i.e., thrombin and FXIIa. Coagulation imbalance is probably caused by abnormally elevated transferrin, which blocks AT’s inactivation effect on coagulation proteases by binding to AT and also interacts with and potentiates thrombin/FXIIa in addition to normal interaction with fibrinogen, thus inducing hypercoagulability.

## Materials and methods

### Specimens of human atherosclerotic plaque and plasma

The Institutional Review Board of the Kunming Institute of Zoology (KIZ) and the Yan’an Affiliated Hospital of Kunming Medical University approved this study (KIZ-YA-20150109). All human specimens were collected with the informed consent of patients prior to the study. Plasma samples from patients with CHD (*n* = 120) and from healthy controls (*n* = 120) were collected from Yan’an Affiliated Hospital of Kunming Medical University (Supplementary information, Table S1). In total, 120 subjects with CHD exhibiting angiographically visible luminal narrowing were selected in the present study. Patients with clinical features of angina pectoris were further diagnosed by coronary angiography. The 120 patients were matched with 120 healthy volunteers as a normal control group with angiographically normal coronary arteries, and no history of hypertension, diabetes mellitus, or hypercholesterolemia disease. Immediately following the blood drawing (with 1.5% EDTA-Na_2_ was utilized as an anticoagulant agent), plasma was obtained by centrifugation at 3000 rpm for 20 min at 4 °C and stored at −80 °C after being sub-packed.

Atherosclerotic plaque specimens were obtained from coronary endarterectomy, and normal arteries were obtained from coronary artery bypass surgery (*n* = 12, age 30–80 years) (Supplementary information, Table S2) from Yan’an Affiliated Hospital of Kunming Medical University. Immediately following surgical removal, some specimens were minced and homogenized at 4 °C for protein extraction, then stored at −80 °C after being sub-packed; some were fixed in 10% buffered formalin to prepare a frozen sections; and some were placed in RNAlater (R0901-500ML, Sigma, USA) for RNA extraction and stored at −80 °C until further use.

### Animals and ethics statement

All animal experiments were approved by the Animal Care and Use Committee at the Kunming Institute of Zoology (SMKX-2016013) and conformed to the US National Institutes of Health’s Guide for the Care and Use of Laboratory Animals (The National Academies Press, 8th Edition, 2011). *Apoe*^*−/−*^ mice (females, 8 weeks old, C57BL/6J background, number of backcrosses: 10 times) and C57BL/6J mice (females, 8 weeks old) were purchased from Vitalriver Experiment Animal Company (Beijing, China) and housed in a pathogen-free environment. Mice were maintained in sterile isolators with autoclaved food and water under 12 h light–12 h dark cycle at 24 °C.

### Chromogenic assays

The effects of CHD patient and healthy control plasma (blood was prepared by mixing a 1:9 volume of trisodium citrate (0.13 M) and blood, with plasma then obtained by centrifugation at 3000 rpm for 20 min at 4 °C) on proteases (kallikrein, FXIIa, FXIa, FVIIa, and thrombin) involved in coagulation were tested using corresponding chromogenic substrates. The tested enzyme was incubated with the plasma (1 μL) in 60 μL Tris–HCl buffer (50 mM, pH 7.4) for 5 min, with the addition of a certain concentration of chromogenic substrate then added, as described below. Absorbance at 405 nm was monitored immediately, and a kinetic curve was recorded using an enzyme-labeled instrument (Epoch, BioTek, USA) for 30 min. Relative enzyme activity was obtained by calculating the enzymatic hydrolysis velocity of its substrate. Human α-thrombin (20 nM, T6884, Sigma, USA) and human α-FXIIa (20 nM, HFXIIa 1212a, Enzyme Research Laboratories, USA) were reacted with 0.2 mM chromogenic substrates of *H-D*-Phe-Pip-Arg-*p*Na·2HCl (CS-01, Hyphen Biomed, France) and *H-D*-Pro-Phe-Arg-*p*NA·2HCl (CS-31, Hyphen Biomed, France), respectively. The concentration used for kallikrein (HPKa 1303, Enzyme Research Laboratory, USA) and FXIa (HFXIa 1111a, Enzyme Research Laboratory, USA) was 40 and 10 nM, respectively, and the corresponding chromogenic substrates were 0.2 mM *H-D*-Pro-Phe-Arg-*p*NA·2HCl (CS-31, Hyphen Biomed, France) and 0.2 mM pyroGlu-Pro-Arg-pNA·HCl (S-2366, Chromogenix, USA), respectively. The concentration used for FVIIa (HFVIIa, Enzyme Research Laboratory, USA) was 20 nM, and the chromogenic substrate was 0.1 mM CH_3_SO_2_-D-CHA-But-Arg-*p*NA·AcOH (ADG217L, Sekisui Diagnostics, Germany).

### Purification and identification of transferrin

Albumin and IgG were first removed using a HiTrap Albumin and IgG depletion column (GE, USA). Plasma was diluted (1:1) with 20 mM Tris–HCl buffer containing 20 mM NaCl (pH 7.8) and then applied to a Resource Q column (17-1177-01, GE, USA) for purification in a fast protein liquid chromatography (FPLC) system (GE, USA). The column was pre-equilibrated with solvent A (20 mM Tris–HCl, pH 7.8) and the elution was performed with a linear gradient of 0–100% solvent B (20 mM Tris–HCl, 1 M NaCl, pH 7.8) over 100 min. The fraction showing potentiation against thrombin and FXIIa was subjected to a Mono Q column (17-5166-01, GE, USA) for further purification using the same elution system as above.

The purified thrombin/FXIIa potentiator (10 μg) was dissolved in 25 mM NH_4_HCO_3_ buffer and reduced by 10 mM dithiothreitol (DTT) for 1 h at 37 °C. The reduced sample was alkylated by 30 mM iodoacetamide dissolved in the same buffer for 30 min at room temperature in a dark place. The alkylation reaction was ended by adding additional DTT. The sample was treated with 1% trypsin (w/w) at 37 °C overnight for mass spectrometry (MS). A matrix-assisted laser desorption ionization time-of-flight (MALDI-TOF/TOF) mass spectrometer (Autoflex speed, BrukerDaltonics, Germany) was employed for data acquisition according to the manufacturer’s instructions. The MS and MS/MS spectra were collected and processed using FlexControl, FlexAnalysis, and BioTools software (BrukerDaltonics, Germany).

### Transferrin measurement in human plasma and tissue specimens

The concentration of transferrin in the plasma of CHD patients and healthy controls was determined using a human transferrin ELISA kit (EK12012-96T, Multi Sciences, China) according to the manufacturer’s instructions. TIBC in plasma of these CHD patients and healthy controls was determined by TIBC kit (ab239715, Abcam, USA) according to the manufacturer’s instructions. The amounts of transferrin in plasma and plaque homogenates were also determined by western blot analysis. Briefly, plasma and tissue homogenates were first separated by 12% sodium dodecyl sulfate–polyacrylamide gel electrophoresis (SDS–PAGE), and then transferred to polyvinylidene difluoride (PVDF) membranes. An anti-transferrin antibody (1:2000, 11019-RP02, Sino Biological Inc, China) was used for immunoreactivity. To control for plasma loading and transfer, membranes were stained by Red Ponceau after transfer, or blotting for β-actin as a plaque homogenates loading control.

### Effects of transferrin on enzymatic activity of coagulation factors

The effects of apo-transferrin or holo-transferrin (Sigma, purity greater than 98%, no residual enzyme activity of FXIIa and thrombin) on proteases involved in coagulation (kallikrein, FXIIa, FXIa, FVIIa and thrombin) were tested using corresponding chromogenic substrates as described above. Human serum albumin (HSA, purity greater than 98%) was used as a control. The effect of transferrin on thrombin to hydrolyze its natural substrate (fibrinogen) was analyzed by reverse phase high performance liquid chromatography (RP-HPLC) system. Briefly, human α-thrombin (0.1 NIH unit) in 40 μL of Tris–HCl (25 mM, pH 7.4) was incubated with 500 μL of fibrinogen (10 mg/mL, 16088, Cayman, USA) in the same buffer containing 0.15 M NaCl in the presence of apo-transferrin (0.2–5 μM) for 30 min at 37 °C. After the incubation, 500 μL of 20% trichloroacetic acid (TCA) was added to stop the reaction, followed by centrifugation at 12,000 rpm for 10 min at 4 °C to precipitate insoluble protein. Aliquots (700 μL) of the supernatant were used for RP-HPLC analysis. The elution system consisted of solvent A (0.025 M ammonium acetate, pH 6.0) and a linear gradient of 0–100% solvent B (50% acetonitrile in 0.05 M ammonium acetate, pH 6.0) over 100 min. Release of FbpA and FbpB was quantified by calculating the corresponding eluted peak area on a C_18_ column (30 cm × 0.46 cm, Hypersil BDS, USA), respectively.

The effect of transferrin on FXIIa to hydrolyze its natural substrate (prekallikrein, PK) was assayed by SDS–PAGE. PK (10 μg, HPK1302, Enzyme Research Laboratory, USA) was incubated with human α-FXIIa (20 nM) in 40 μL of Tris–HCl buffer (50 mM, pH 7.4) in the presence of apo-transferrin (0.2–5 μM). After 30 min of incubation at 37 °C, all reactions were applied to 12% SDS–PAGE. The production of kallikrein heavy chain (HC ∼52 kDa) and light chain (LC ∼ 36 and 33 kDa) was detected by western blot analysis using anti-plasma PK polyclonal antibody (1:1000, SAPK-IG, Enzyme Research Laboratories, USA). In addition, PK, FXIIa heavy chain (FXIIa HC), and transferrin were also detected using anti-plasma PK, anti-FXII (1:2000, ab242123, Abcam, USA), and anti-transferrin (1:2000, 11019-RP02, Sino Biological Inc, China) antibodies, respectively. The HC of kallikrein was quantified using ImageJ software. Transferrin’s effects on prothrombin (HP 1002, Enzyme Research Laboratories, USA) and FXII (HFXII 1212, Enzyme Research Laboratories, USA) activation were assayed using the corresponding chromogenic substrates and reaction system as described above.

### Effects of transferrin on thrombin/FXa–AT complex formation in vitro

Briefly, transferrin (2.5–10 μM), AT (2 μM, A2221-125UG, Sigma, USA), and thrombin (20 nM) or FXa (20 nM, HFXa 1011, Enzyme Research Laboratory, USA) were incubated simultaneously in 60 μL of Tris–HCl buffer (50 mM, pH 7.4) for 5 min at 37 °C, with thrombin or FXa activity then tested using corresponding chromogenic substrates as described above for thrombin and Z-D-Arg-Gly-Arg-pNA·2HCl (S-2765, Aglyco, China) for FXa. Thrombin or FXa only or incubated with AT in the same buffer were also used to test thrombin and FXa activity. Equal concentrations of HSA were used as a control. The thrombin–AT complex (TAT) was tested using an ELISA kit (ab108907, Abcam, USA) according to the manufacturer’s instructions. The FXa–AT complex was measured using sandwich ELISA. Briefly, plates were coated with anti-human FXa antibody (PAB19898, Abnova, USA) and blocked with 2% bovine serum albumin (BSA) before incubation with the above reaction solution at 37 °C. After thrice washing with PBST (PBS (80 mM Na_2_HPO_4_, 1.5 M NaCl, 20 mM KH_2_PO_4_, and 30 mM KCl), 0.5% Tween-20, pH 7.4) at room temperature, the FXa–AT complex was detected following incubation with HRP-conjugated anti-human AT antibody (1:200, SAAT-APHRP, Enzyme Research Laboratory, USA) at 37 °C. Relative levels of TAT and FXa–AT complex were calculated.

### Effects of transferrin on blood coagulation

Healthy human plasma was collected from the Kunming Blood Center, and blood was prepared by mixing a 1:9 volume of trisodium citrate (0.13 M) and blood, with plasma then obtained by centrifugation at 3000 rpm for 20 min at 4 °C. To test the effects of transferrin on plasma recalcification time, 20 μL of plasma was incubated with human apo- or holo-transferrin (0.2–5 μM) in 60 μL of HEPES buffer (20 mM HEPES, 150 mM NaCl, pH 7.4) for 10 min at 37 °C, followed by the addition of 60 μL of 25 mM CaCl_2_ preheated at 37 °C. Clotting was monitored at 650 nm and clotting time was calculated by measuring the time to half maximal increase in absorbance.

### Mouse AS model

*Apoe*^*−/−*^ mice (females, 8 weeks old) were fed either a high fat diet (HFD, 21% fat, 0.15% cholesterol) for 6 weeks to induce AS or a normal diet (ND) to serve as the controls. After 6 weeks, mice were sacrificed and blood was collected for further plasma preparation (blood was prepared by mixing a 1:9 volume of trisodium citrate (0.13 M) and blood, with plasma then obtained by centrifugation at 3000 rpm for 20 min at 4 °C). Following surgical removal, the aortic root was immediately fixed in 4% paraformaldehyde dissolved in PBS at 4 °C overnight. Organs, including the liver, brain, spleen, muscle, kidney, and stomach, and plaque were also collected and placed in RNA later for RNA extraction or PBS for protein extraction. The aortic root specimens were cut into 8-μm sections using a freezing microtome (Thermo Scientific, USA). Some sections of aortic root were stained with oil-red O to investigate atherosclerotic lesions, whereas others were used for immunofluorescent assay, as described below. Images of oil-red O staining were acquired by a dark field microscope (Life technologies, USA), and the oil-red O-stained plaque areas were measured by Image J.

The RNA extraction and cDNA reverse transcription procedures were performed by using an RNA extraction kit (DP419, Tiangen, China) and reverse transcription kit (A5000, Promega, USA), respectively, in accordance with the manufacturer’s instructions. Transferrin expression was quantified by both qRT-PCR (forward primer (5′–3′): GGACGCCATGACTTTGGATG; reverse primer (5′–3′): GCCATGACAGGCACTAGACC) and western blot analysis. PCR was performed on a CFX-96 Touch Real-Time Detection System (Bio-Rad, USA). The concentration of transferrin in mouse plasma was determined using a mouse transferrin ELISA kit (ab157724, Abcam, USA). The amount of transferrin in plaque homogenates was also determined by western blot analysis using the anti-transferrin antibody (1:2000, 11019-RP01, Sino Biological Inc, China), as described above.

### Surface plasmon resonance (SPR) analysis

BIAcore 2000 (GE, USA) was used to analyze the interaction between transferrin and clotting factors using HSA as a control. Briefly, apo-transferrin or holo-transferrin was first diluted (20 μg/mL) with 200 μL of sodium acetate buffer (10 mM, pH 5) and then flowed across the activated surface of a CM5 sensor chip (BR100012, GE, USA) at a flow rate of 5 μL/min, reaching a resonance unit (RU) of ~ 2000. The remaining activated sites on the chip were blocked with 75 μL of ethanolamine (1 M, pH 8.5). Serial concentrations of thrombin, FXIIa, fibrinogen, AT, prothrombin, or FXII in Tris–HCl buffer (20 mM, pH 7.4) were applied to analyze their interactions with immobilized apo-transferrin or holo-transferrin at a flow rate of 10 μL/min. The *KD* for binding as well as the *Ka* and *Kd* rate constants were determined using the BIA evaluation program (GE, USA). The CM5 sensor chip was coupled with 100 RU of transferrin, using the method above. The RU change is reported in BIAcore technology where a 1000 RU response is equivalent to a change in surface concentration of about 1 ng/mm^2^ of protein and the binding molar ratio between transferrin and fibrinogen or AT was calculated by comparing the saturation RU of the flowing phase and RU of the immobilized phase, as the method described.^53^

### Native PAGE

Basic native PAGE was used to further analyze the interaction between transferrin and clotting factors. In brief, human apo-transferrin (8 μg) was first incubated with different concentrations of human thrombin or human FXIIa (2–8 μg) in 30 μL of Tris–HCl buffer (50 mM, pH 7.4) for 10 min at 37 °C, and then applied to an 8% precast gel (PG00810-N, Solarbio, China) to analyze complex formation between transferrin and thrombin/FXIIa in running buffer (0.05 M Trizma, 0.38 M glycine, pH 8.9) at 200 V constant voltage for 1 h. Fibrinogen (8 μg) or AT (8 μg) and different concentrations of apo-transferrin (2–8 μg) were also used to analyze the interaction by basic native PAGE. Staining analysis was performed with staining solution (0.25% (v/v) Coomassie Brilliant Blue R containing 50% (v/v) methanol, 40% (v/v) dH_2_O and 10% (v/v) acetic acid) and the destaining solutions (mainly consists of 15% (v/v) methanol, 10% (v/v) acetic acid and 75% (v/v) dH_2_O).

### Confocal microscopy

Human atherosclerotic plaque or mouse aortic root specimens were first cut into 8-μm sections using a freezing microtome (Thermo Scientific, USA). For immunofluorescence detection of transferrin, thrombin, or FXIIa in the atherosclerotic plaque, the sections were first incubated with anti-mouse transferrin (1:200, 11019-RP01), anti-human transferrin (1: 200, 11019-RP02), anti-thrombin (1:200, ab17199, Abcam, USA), and anti-mouse or human FXII (1:200, SC-6670 or SC-66752, Santa Cruz, USA) antibody at 4 °C overnight, respectively. After washing three times with PBS to remove the excess primary antibody, the section was incubated with a fluorescently labeled secondary antibody for 1 h at 37 °C. Cell nuclei were stained with 4, 6-diamidino-2-phenylindole (DAPI, P36941, Lifetechnologies, USA). Images of immunofluorescence were obtained using an Olympus FluoView 1000 confocal microscope according to the manufacturer’s instructions.

### APTT and PT assays

For the APTT assay, 50 μL of APTT reagent (F008-1, Nanjing Jiancheng Bioengineering Institute, China) was incubated with 50 μL of plasma for 3 min at 37 °C, followed by the addition of 50 μL of CaCl_2_ (25 mM) preheated at 37 °C to test the clotting time, with absorbance monitored at 405 nm using a semi-automatic coagulation analyzer (ThromboScreen 400c, Pacific hemostasis, USA). To test PT, 50 μL of plasma preheated at 37 °C was mixed with 100 μL of PT reagent (F007, Nanjing Jiancheng Bioengineering Institute, China) preheated at 37 °C for 15 min, with absorbance monitored at 405 nm.

### Bleeding time measurement

A mouse tail transection model was used to test tail bleeding time. In brief, mouse tails were cut 2 mm from the tip, and then carefully immersed in 20 mL of saline warmed to 37 °C. Bleeding time was recorded until blood flow ceased.

### Molecular docking and inhibitory peptides designing

Transferrin was docked into the exosite I of human thrombin. The human thrombin model was homologically constructed from known structures (PDB ID: 4NZQ). With the assistance of another two complex structures with PDB ID: 1A2C and 1HAH, the binding site was locally placed for further docking processes. Meanwhile, two structures (PDB ID: 4XE4 and 4XDE) were used to build the working model of human FXIIa. The FXIIa model was aligned along the thrombin model to confirm the possible groove-like binding area. The human transferrin model was then extracted from the complex (PDB ID: 3V8X) and cleaned and reconstructed by repairing the missing residues. The above three models were all optimized by short molecular dynamics to eliminate steric clashes by side chain packing. The modified transferrin model was docked into exosite I site of human thrombin and a similar site of FXIIa by the alignment of these two structures. The docking processes were performed with a standard pipeline protocol of Discovery Studio (version 3.1). During the docking process, common parameters were used to obtain more accurate results and ZRank scored the top 2000 poses with RMSD cutoff of 10.0 Angstroms.

Based on molecular docking analysis of transferrin and thrombin/FXIIa, two peptides (TH16 and FX18) were characterized and synthesized. TH16 (RIGKHSRTRYERNIEK) and FX18 (RRNHSCEPCQTLAVRSYR) were deduced from the sequence of the exosite I domain of thrombin (GenBank number (NM_000506.3)) and the sequence of the exosite I domain analogue of FXIIa (GenBank number (NP_000496.2)), respectively. The scrambled peptides of TH16 (TH16-scr, RKKGIRRYTERHSNIE) and FX18 (FX18-scr, SCPTHYSRQRCRNAVLER) were also designed and synthesized.

### Effects of transferrin interferences on mouse AS development

Several methods of transferrin interferences, including lentivirus or retrovirus (10^7^ transducing units) injection into the tail vein of *Apoe*^*−/−*^ mice to induce transferrin overexpression or knockdown, transferrin antibody intravenous injection twice (50 μg per time) per week for 6 weeks, and inhibitory peptides (TH16 and FX18) intravenous injection under different dosages (0.2–5 mg/kg) thrice per week for 3 weeks, were performed to evaluate their effects on the development of mouse AS induced by HFD, as described above. All transferrin interferences were started from the beginning of HFD induction. According to the methods above, tail bleeding time, FeCl_3_-induced carotid artery thrombus formation, plasma thrombin and FXIIa generation, APTT, and PT were also evaluated in these mouse models. The amounts of iron and ferritin in the plasma were determined using an iron test kit (ferrozine method, TC1016-100T, Leagene, China) and ferritin test kit (SEA518Mu, Uscn, China), respectively. Mean corpuscular volume (MCV), mean corpusular hemoglobin (MCH), and mean corpuscular hemoglobin concentration (MCHC) were assayed using a blood routine test machine (BC-2800Vet, Mindray, China). Tail bleeding time, FeCl_3_-induced carotid artery thrombus formation, plasma thrombin and FXIIa generation, APTT, or PT were also evaluated in C57BL/6J mice that performed transferrin interferences (transferrin overexpression, transferrin knockdown, anti-transferrin antibody administration, and interference peptides TH16 and FX18 administration).

### FeCl_3_-induced carotid artery thrombus formation

The C57BL/6J mice (females, 8 weeks old) and *Apoe*^*−/−*^ mice (females, 8 weeks old) with transferrin interference were first anaesthetized with 2.0% isoflurane and core body temperature was maintained at 37 °C during the whole surgery. One of the carotid arteries was exposed by cervical incision, and separated from the adherent tissue and vagus nerve. Thrombosis was induced by applying a piece (2 × 2 mm) of filter paper that was pre-soaked with 10% (w/v) FeCl_3_ solution to the exposed carotid artery. Blood flow of the carotid arteries from all groups was measured by laser speckle perfusion imaging (PeriCam PSI, HR, Sweden) at 5 min and 10 min after FeCl_3_ induction, respectively. The perfusion unit of region of interest (ROI) was also recorded to quantify the blood flow changes.

### Statistical analysis

The data obtained from independent experiments are presented as the mean ± SD. All statistical analyses were two-tailed and with 95% confidence intervals (CI). Results were analyzed using one-way ANOVA with Dunnett’s post hoc test or unpaired t-test using Prism 6 (GraphPad Software) and SPSS (SPSS Inc, USA). Differences were considered significant at *P* < 0.05.

For more detailed information, please see Supplementary information, Data S1.

## Supplementary information

Supplementary information, Data S1

Supplementary information, Figure S1

Supplementary information, Figure S2

Supplementary information, Figure S3

Supplementary information, Figure S4

Supplementary information, Figure S5

Supplementary information, Figure S6

Supplementary information, Figure S7

Supplementary information, Figure S8

Supplementary information, Figure S9

Supplementary information, Figure S10

Supplementary information, Table S1

Supplementary information, Table S2

Supplementary information, Table S3

Supplementary information, Table S4
