# Supplementary material for: Transferrin plays a central role in coagulation balance by interacting with clotting factors
Source: Cell Res. 2019 Dec 6;30(2):119–32. doi: 10.1038/s41422-019-0260-6 (PMC7015052; doi:10.1038/s41422-019-0260-6)
Supplement: Supplementary file 1 — Supplementary information, Data S1 [file 41422_2019_260_MOESM1_ESM.pdf]

Materials and Methods

Measurement of prothrombin and FXII concentration

Proteins in plasma from coronary heart disease (CHD) patients and healthy controls were first separated by sodium dodecyl sulfate-polyacrylamide gel electrophoresis (SDS-PAGE) with 12% gel concentration, and then transferred to polyvinylidene difluoride (PVDF) membranes. Membranes were blocked with 5% non-fat dried milk (BD, USA) dissolved in TBST buffer (2.42 g/L Tris base, 8 g/L NaCl, 0.1% Tween-20, pH 7.6) for 2 h at room temperature. After thrice washing with TBST buffer, the PVDF membranes were incubated with primary antibody at 4 ℃ overnight, and then incubated with secondary antibody for another 1 h at room temperature. After washing with TBST, the membrane was developed with an enhanced chemiluminescence kit (PA112, Tiangen, China) by ImageQuant LAS 4000 mini (GE Healthcare, USA). Two primary antibodies (mouse polyclonal antibody against thrombin (1:2000, ab17199, Abcam, USA) and goat polyclonal antibody against FXII (1:2000, SC-66752, Santa Cruz, USA)) were used to detect prothrombin and FXII, respectively. To control for plasma loading and transfer, membranes were stained by Red Ponceau after transfer.

Effects of transferrin on thrombin-induced platelet aggregation

Platelets from healthy volunteers were collected from the Kunming Blood Center, Yunnan Province, China. The platelets were thrice washed with Tyrode's buffer (12 mM $NaHCO_3$, 138 mM NaCl, 5.5 mM glucose, 2.9 mM KCl, 2 mM $MgCl_2$, 0.42 mM NaH_2PO_4, 10 mM HEPES, pH 7.4), then re-resuspended in the same buffer. Platelets

(300 μl, 1 × 10^9 cells/ml) were incubated with transferrin (0.2–5 μM) for 5 min at 37 ℃, and 1 NIH unit of human α-thrombin (T6884, Sigma, USA) was then added to induce platelet aggregation. Light transmittance was monitored immediately, and a kinetic curve was recorded using a platelet aggregation instrument for 5 min (LBY-NJ4, Precil, China). The maximum platelet aggregation rate (MPAR) was calculated.

Recombinant expression of transferrin, thrombin, and their mutants

The prokaryotic expression vector was constructed by inserting the DNA sequence encoding mature transferrin (GenBank: AAA61140.1, 679 amino acids) or transferrin mutant (E333R and E338R) between the KpnI and XhoI sites of the pET-32a (+) vector. DNA encoding thrombin (NM_000506.3, 308 amino acids) or thrombin exosite I mutant (R117A and R122A) was inserted between the BamHI and XhoI sites of the pSmart-I vector. The vectors were transformed into *Escherichia coli* (*E. coli*) Rosetta (DE3), which was induced by 0.8 mM isopropyl β-D-thiogalactoside (IPTG) for 6 h in a 110-rpm shaker at 28 ℃. After induction, the *E. coli* cells were collected by centrifugation at 12,000 rpm/min for 15 min at 4 ℃, and resuspended in the binding buffer (20 mM Tris-HCl, 500 mM NaCl, 5 mM imidazole, pH 7.4). The cells were then homogenized using an Ultrasonic Cell Disruption System (XINYI-IID, XinYi, China). The supernatant was collected by centrifugation at 12,000 rpm/min for 2 h at 4 ℃. A Ni^{2+} affinity chromatography column was equilibrated in advance with the binding buffer. The collected supernatant containing the fusion protein was subsequently loaded on the Ni^{2+} affinity chromatography column at a flow rate of 0.7

ml/min. The bound fusion proteins were eluted with five column volumes of elution buffer (20 mM Tris-HCl, 250 mM NaCl, 500 mM imidazole, pH 7.4). The eluted fraction was resuspended in 20 mM Tris-HCl, pH 7.8, and the salt was removed using an ultrafiltration device (UFC500324, Millipore, USA).

Enterokinase (9014-74-8, C&M Biolabs, USA) was added to the reaction buffer (10 mM Tris, 10 mM $CaCl_{2,}$ pH 8.0) and maintained for 24 h at 37 ℃ to release the recombinant transferrin. For the release of recombinant thrombin, small Ubiquitin-Like Modifier (SUMO) protease (1 unit, 12588-018, Lifetechnologies, USA) was added to the reaction buffer (50 mM Tris-HCl, 0.2% Igepal, 1 mM dithiothreitol (DTT), pH 8.0) and maintained for 16 h at 4 ℃. Hydrolyzed recombinant proteins were purified by a Mono Q^{TM} 5/50 GL column (17-5166-01, GE, USA) on a fast protein liquid chromatography (FPLC) system. The column was pre-equilibrated with solvent A (20 mM Tris-HCl, pH 7.8) and elution was performed with a linear gradient of 0–100% solvent B (20 mM Tris-HCl, 1 M NaCl, pH 7.8) over 100 min.

Molecular docking combined with Molecular Dynamics (MD) and binding free energy calculation

To model the transferrin-antithrombin (AT) complex, we used the known structure of transferrin and AT for protein docking. Crystal structure of transferrin (PDB ID: 3V83) was docked to the structure of AT (PDB ID: 2GD4) as the method described. MD simulation of AT and transferrin were performed to evaluate the stability and conformational changes. All MD simulations were performed using

Amber99SB-ILDN force field[1] by GROMACS 5.1 package[2], and running on high-performance Linux system (National Supercomputing Center in Shenzhen, China). During MD simulations, all the systems were solvated using TIP3P[3] water model in a periodic box, followed by addition of 6 Na^+ ions to neutralize the systems. Before MD simulations, energy minimization, NVT, and NPT equilibration were performed. Finally, MD simulations were run for 20 ns time scale under constant temperature (300 K) and pressure (1 atm). Snapshots were collected from the end of MD simulations and PDBs were generated (Supplementary information, Fig. S7c). The binding free energies and contribution of residues to the binding energy of the complexes between AT and transferrin were analyzed during equilibrium phase from 5 to 20 ns MD simulations using the g_mmpbsa tool[4]. Based on the MD analysis, peptide (RCL19) was characterized and synthesized. RCL19 (EAAASTAVVIAGRSLNPNR) was deduced from the sequence of the reactive center loop (RCL) of AT, and the scrambled peptide of RCL19 (RCL19-scr, GANPRELNSAATIASVARV) was also designed and synthesized. Interaction between transferrin and RCL19 or RCL19-scr was analysed by surface plasmon resonance (SPR) method as described.

Crosslinking assay

Bis (sulfosuccinimidyl) suberate (BS^3, 1 mM, 21586, Thermo, USA) was used to crosslink plasma (1 μl, CHD patients and normal controls) in HEPES buffer (20 mM, pH 7.4) for 30 min at room temperature. Tris-HCl (10 mM, pH 7.5) was then used to stop the reaction. The crosslinked plasma samples were subjected to 12% SDS-PAGE

separation. A rabbit polyclonal antibody against transferrin (1:2000, 11019-RP02, Sino Biological, Inc. China) was used to detect transferrin. After stripping the PVDF with the stripping buffer (PH1498, Phygene, China), anti-thrombin (1:2000, ab17199, Abcam, USA) and anti-FXII (1:2000, SC-66752, Santa Cruz, USA) antibodies were used to detect prothrombin and FXII, respectively. To control for plasma loading and transfer, membranes were stained by Red Ponceau after transfer. Typical tissue specimens (AS patients, and normal controls) were also crosslinked by BS^3 followed by 12% SDS-PAGE to analyze the transferrin-prothrombin/FXII complexes.

Immunoprecipitation

Anti-transferrin antibody (2 μg) and normal human plasma (1 μl) were first mixed and incubated for 16 h at 4 ℃ in 30 μl of Tris-HCl buffer (25 mM, pH 7.4). Protein A agarose (20 μl, P2006, Beyotime, China) was then added to couple with the anti-transferrin antibody and incubated for 3 h at 4 ℃. After centrifugation at 2500 rpm for 5 min at 4 ℃, loading buffer (10 μl, 4 × CW0027A, CWBIO, China) was added and boiled for 10 min to obtain the coupled proteins. All proteins were subjected to 12% SDS-PAGE separation and the polyclonal antibodies against thrombin (1:2000, ab17199, Abcam, USA), FXII (1:2000, SC-66752, Santa Cruz, USA), and transferrin (1: 2000, 11019-RP02, Sino Biological Inc, China) were used to identify prothrombin, FXII, and transferrin, respectively. IgG (2 μg, NI01, Sigma, USA) was used as the control.

Generation of lentiviral and retroviral vectors and virus package for transferrin overexpression or knockdown

The coding region of the mouse transferrin (GenBank: BC092046.1) was synthesized and cloned into pLVX-Puro lentiviral plasmid (Clontech, USA) between the EcoRI and BamHI sites. Empty pLVX-Puro plasmid was used as a blank control. The oligonucleotides of the shRNA sequence targeting transferrin (sh-Tf) and its scrambled control shRNA (sh-scr) sequence were synthesized by Sangon Biotech (Shanghai, China). The sequence of sh-Tf is 5'-AAGAATCTGAAGCAGGAAGAC TTCAAGAGAGTCTTCCTGCTTCAGATTCTTCTTTTTT-3' (forward oligo), while that for sh-scr is 5'-ACTGAGACTGAACAAAGGAAGTTCAAGAGACTTCCTTTGTTCAGTCTCAG TCTTTTTT-3' (forward oligo). The oligonucleotides of sh-Tf and sh-scr including the digested sites of BamHI and EcoRI were annealed with their complementary sequences and inserted into the BamHI and EcoRI sites of the RNAi-Ready pSIREN-RetroQ retroviral vector (Clontech, USA) to form retroviral vectors of psh-Tf and psh-scr, respectively. The lentiviral vector for transferrin overexpression and retroviral vector for transferrin knockdown were transfected into murine embryonic liver cells (BNL CL.2 cells, Conservation Genetics CAS Kunming Cell Bank, China) to test the efficiency of transferrin overexpression or knockdown using western blot and qRT-PCR. The primers for PCR were forward primer (5'-3'): GGACGCCATGACTTTGGATG and reverse primer (5'-3'): GCCATGACAGGCACTAGACC). The transfection procedure was performed with lipofectamine 2000 (11668019, Life technologies, USA) according to the manufacturer's instructions.

HEK 293T cells (Conservation Genetics CAS Kunming Cell Bank, China) and EcoPack™ 2–293 cells (Clontech, USA) were used to package lentiviruses and retroviruses, respectively. For each virus preparation, 1.5 $\times 10^7$ HEK 293T cells or EcoPack™ 2–293 cells were seeded in a T75 flask for 24 h before transfection. The pLVX-Puro empty lentiviral vector (20 μg) or with mouse transferrin gene and packaging plasmids, including psPAX2 (15 μg, Addgene Inc., USA) and pMD2G (10 μg, Addgene Inc., USA), were co-transfected into HEK 293T cells by lipofectamine 2000. Each flask of EcoPack™2–293 cells was transfected with 25 μg of retroviral vector psh-Tf or psh-scr in 70 μl of lipofectamine 2000. The medium was changed with fresh medium after 8 h transfection, and the supernatants containing viruses were harvested after 48 h from the first transfection and concentrated by ultracentrifugation using a P28S rotor (Himac, Japan) at 25,000 rpm for 3 h at 4 ℃, and then immediately stored at -80 ℃ after sub-packing. The HEK 293T cells (100 μl, 4×10^4 cells) were seeded in a 96-well plates to measure virus titers. Briefly, after infection by six gradients (dilution ratio: 10, 10^2, 10^3, 10^4, 10^5, and 10^6) of lentiviruses (pLVX-Puro-Tf, and negative control) or retroviruses (psh-Tf, and psh-scr) for 48 h, puromycin (2 μg/ml, ab141841, Abcam, USA) was added to screen infected cells for 48 h and virus titer was measured by counting infected living cells.

Anti-transferrin polyclonal antibody preparation

Mouse transferrin (T0523, Sigma, USA) was used to immunize rabbits to produce an anti-transferrin polyclonal antibody. Briefly, rabbits (males, 2 kg) were primed by subcutaneous injections of 500 μg of mouse transferrin in 1 ml of complete Freund's

adjuvant (Sigma, USA) on day 0, followed by subcutaneous booster injections of half doses of the antigens in incomplete Freund's adjuvant (Sigma, USA) on day 14, 28, and 42. The titer and specificity of polyclonal antibodies were screened by indirect ELISA and western blot, respectively. Anti-transferrin polyclonal antibodies were then purified from rabbit serum on a protein G column (Amersham Biosciences, Piscataway, NJ, USA).

Measurement of thrombin and FXIIa generation of mouse plasma

Plasma samples from transferrin-overexpressed, knockdown or anti-transferrin antibody-treated mice were used in this assay. Briefly, plasma (80 μl) was added to 96-well microtiter plates, followed by the addition of 20 μl of HEPES buffer (20 mM HEPES, 150 mM NaCl, 60 mg/ml BSA, pH 7.4) containing tissue factor (1 pM) (ab87476, Abcam, USA) and phospholipids (4 μM) (20 mol% phosphatidylethanolamine, 20 mol% phosphatidylserine and 60 mol% phosphatidylcholine, Sigma, USA). Thrombin fluorogenic substrate (20 μl, 0.8 mM, Z-Gly-Gly-Arg-AMC, Bachem, Switzerland) containing 20 mM $CaCl_2$ was then added to start the reaction. Fluorescence was measured at 30 s intervals over 30 min using a microtiter plate fluorometer (Ascent reader, Helsinki, Finland). The excitation and emission wavelengths were 390 and 460 nm, respectively. Relative thrombin activity was calculated by comparing fluorescence values. For plasma FXIIa generation tests, briefly, plasma (80 μl) was mixed with 20 μl of HEPES buffer, followed by 20 μl of FXIIa fluorogenic substrate (0.8 mM, Boc-Gln-Gly-Arg-AMC, Bachem, Switzerland) containing 20 mM $CaCl_2$ and 40 μg/ml collagen (C9879,

Sigma, USA) to start the reaction. Relative FXIIa activity was calculated by comparing fluorescence values.

Supplementary References

1. Lindorff-Larsen, K., *et al.* Improved side-chain torsion potentials for the Amber ff99SB protein force field. *Proteins* **78**, 1950-1958 (2010).
2. Abraham, M.J., *et al.* GROMACS: High performance molecular simulations through multi-level parallelism from laptops to supercomputers. *SoftwareX* **1-2**, 19-25 (2015).
3. Mahoney, M.W. & Jorgensen, W.L. A five-site model for liquid water and the reproduction of the density anomaly by rigid, nonpolarizable potential functions. *J Chem Phys* **112**, 8910-8922 (2000).
4. Kumari, R., Kumar, R., Lynn, A. & Consort, O.S.D.D. g_mmpbsa-A GROMACS Tool for High-Throughput MM-PBSA Calculations. *J Chem Inf Model* **54**, 1951-1962 (2014).
