# Supplementary material for: Transferrin plays a central role in coagulation balance by interacting with clotting factors
Source: Cell Res. 2019 Dec 6;30(2):119–32. doi: 10.1038/s41422-019-0260-6 (PMC7015052; doi:10.1038/s41422-019-0260-6)
Supplement: Supplementary file 2 — Supplementary information, Figure S1 [file 41422_2019_260_MOESM2_ESM.pdf]

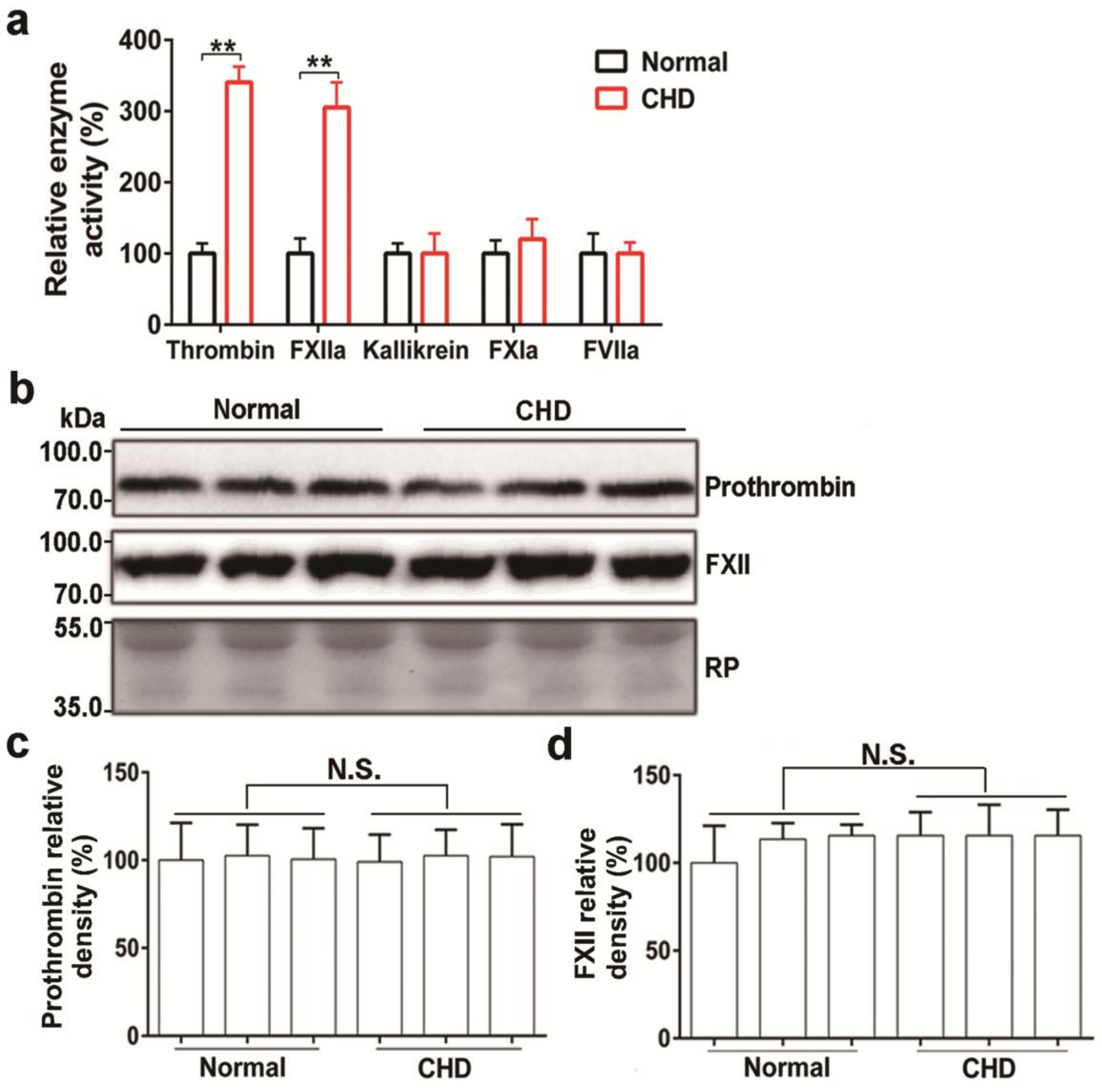

Fig. S1 (a) Effects of plasma from healthy volunteers and CHD patients on enzymatic activities of purified human thrombin, FXIIa, kallikrein, FXIa, and FVIIa by using their corresponding chromogenic substrates, and potentiating activity of transferrin in plasma is dominating under this testing conditions. Western blot **(b)** and quantification analysis of prothrombin **(c)** and FXII **(d)** in plasma of patients. Red Ponceau (RP)-stained blot in panel **b** is the loading control. Data represent mean ± SD (n = 12), $^{**}p < 0.01$ by unpaired t-test. N.S.: no significance.
