# Supplementary material for: Transferrin plays a central role in coagulation balance by interacting with clotting factors
Source: Cell Res. 2019 Dec 6;30(2):119–32. doi: 10.1038/s41422-019-0260-6 (PMC7015052; doi:10.1038/s41422-019-0260-6)
Supplement: Supplementary file 3 — Supplementary information, Figure S2 [file 41422_2019_260_MOESM3_ESM.pdf]

a **b** **c**

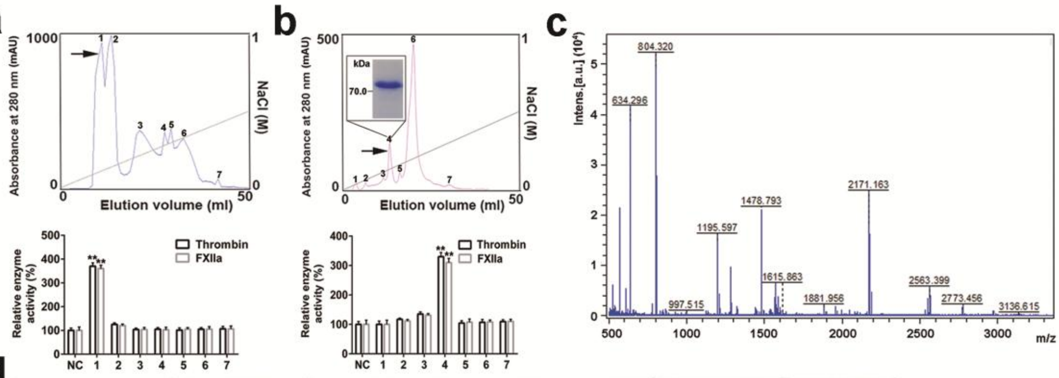

d

Experimental mass	Theoretical mass	Sequence of matched fragment	Fragment position	Modifications
996.5078	996.4698	K.ASYLDCIR.A	62 - 69	
1194.5895	1194.5452	K.DSGFQMNQLR.G	123 - 132	
2170.1557	2170.0881	R.SAGWNIPIGLLYCDLPEPR.K	144 - 162	
1614.8556	1614.8114	K.HSTIFENLANKADR.D	226 - 239	
1880.9485	1880.8687	K.ADRDQYELLCLDNTR.K	237 - 251	
1538.7586	1538.7035	R.DQYELLCLDNTR.K	240 - 251	
2548.3819	2548.2856	R.KPVDEYKDCHLAQVPSHTVVAR.S	252 - 273	
2529.3153	2529.2322	R.SMGGKEDLIWELLNQAQEHFGK.D	274 - 295	
1490.7999	1490.7518	K.SKEFQLFSSPHGK.D	298 - 310	
1322.6890	1322.7095	K.DSAHGFLKVPPR.M	316 - 327	
1477.7853	1477.7275	K.MYLGYEYVTAIR.N	332 - 343	
1493.7744	1493.7224	K.MYLGYEYVTAIR.N	332 - 343	Oxidation (M)
2158.0693	2158.0075	K.IMNGEADAMSLDGGFVYIAGK.C	400 - 420	
1576.8158	1576.8072	R.TAGWNIPMGLLYNK.I	476 - 489	
2970.4051	2970.3099	K.LCMGSGLNLCEPNNKEGYYGYTGAFR.C	516 - 541	
1282.6060	1282.5618	K.EGYYGYTGAFR.C	531 - 541	
1951.9954	1951.9309	K.NLNEKDYELLCLDGTR.K	572 - 587	
1585.8170	1585.7671	R.KPVEEYANCHLAR.A	588 - 600	
1124.6038	1124.6124	K.EACVHKILR.Q	613 - 621	
1564.8553	1564.7919	K.DLLFRDDTVCLAK.L	647 - 659	
1530.7486	1530.6807	K.CSTSSLLEACTFR.R	684 - 696	

e

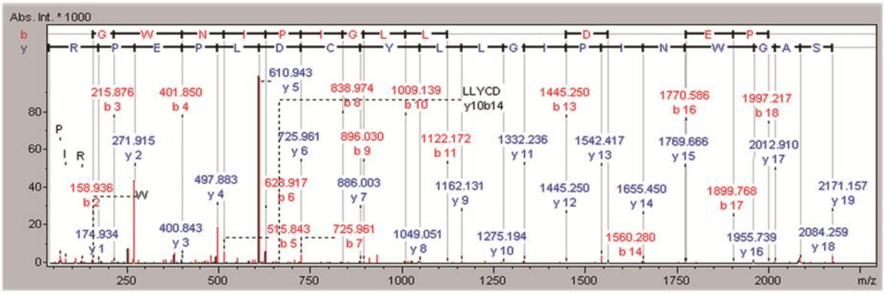

f

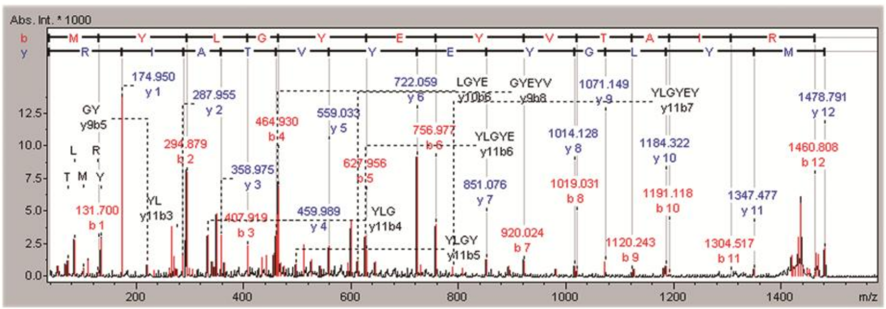

g

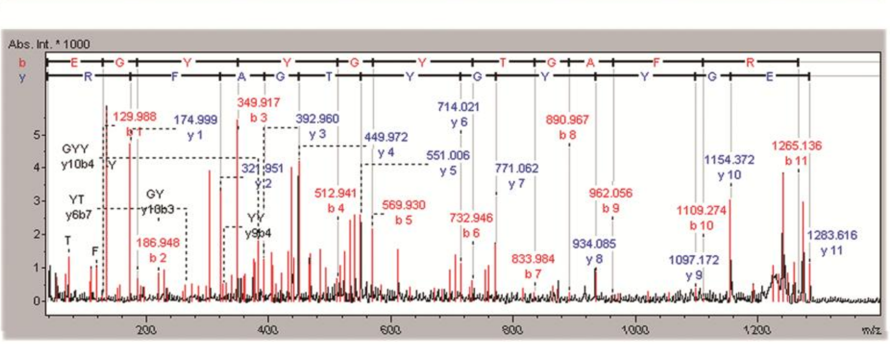

Fig. S2 Purification and identification of transferrin from CHD plasma. (a) FPLC Resource Q chromatogram of atherosclerotic plasma after albumin and IgG depletion (top). Main FPLC fractions were applied to test their effects on enzymatic potentiation of thrombin and FXIIa (bottom). **(b)** Pooled fraction indicated by an arrow in panel **(a)** was further purified by a Mono Q FPLC column (top) and pharmacological tests (bottom). Fraction 4, showing potentiation activity against thrombin and FXIIa, was subjected to 12% SDS-PAGE (inset). Thrombin and FXIIa potentiating peaks in **(a)** and **(b)** are indicated by arrows. **(c)** Analysis of trypsin digested products of purified potentiator of thrombin and FXIIa by MALDI-TOF MS. **(d)** Comparison of experimental molecular mass of peptides resulting from trypsin digestion in "c" with theoretical molecular mass in NCBI and SwissProt databases with Mascot search software and experimental molecular mass is consistent with the theoretical molecular mass of human transferrin in databases. Three peptides produced by trypsin digestion in "c" with m/z of 2171.163 **(e)**, 1478.793 **(f),** and 1283.613 **(g)**, respectively, were selected as parent ions for tandem mass spectrometry, with MS/MS spectra used for sequence analysis by BioTools software (Bruker Daltonics, Germany) and the three peptides sequences are consistent with human transferrin sequence.
