# Supplementary material for: Transferrin plays a central role in coagulation balance by interacting with clotting factors
Source: Cell Res. 2019 Dec 6;30(2):119–32. doi: 10.1038/s41422-019-0260-6 (PMC7015052; doi:10.1038/s41422-019-0260-6)
Supplement: Supplementary file 4 — Supplementary information, Figure S3 [file 41422_2019_260_MOESM4_ESM.pdf]

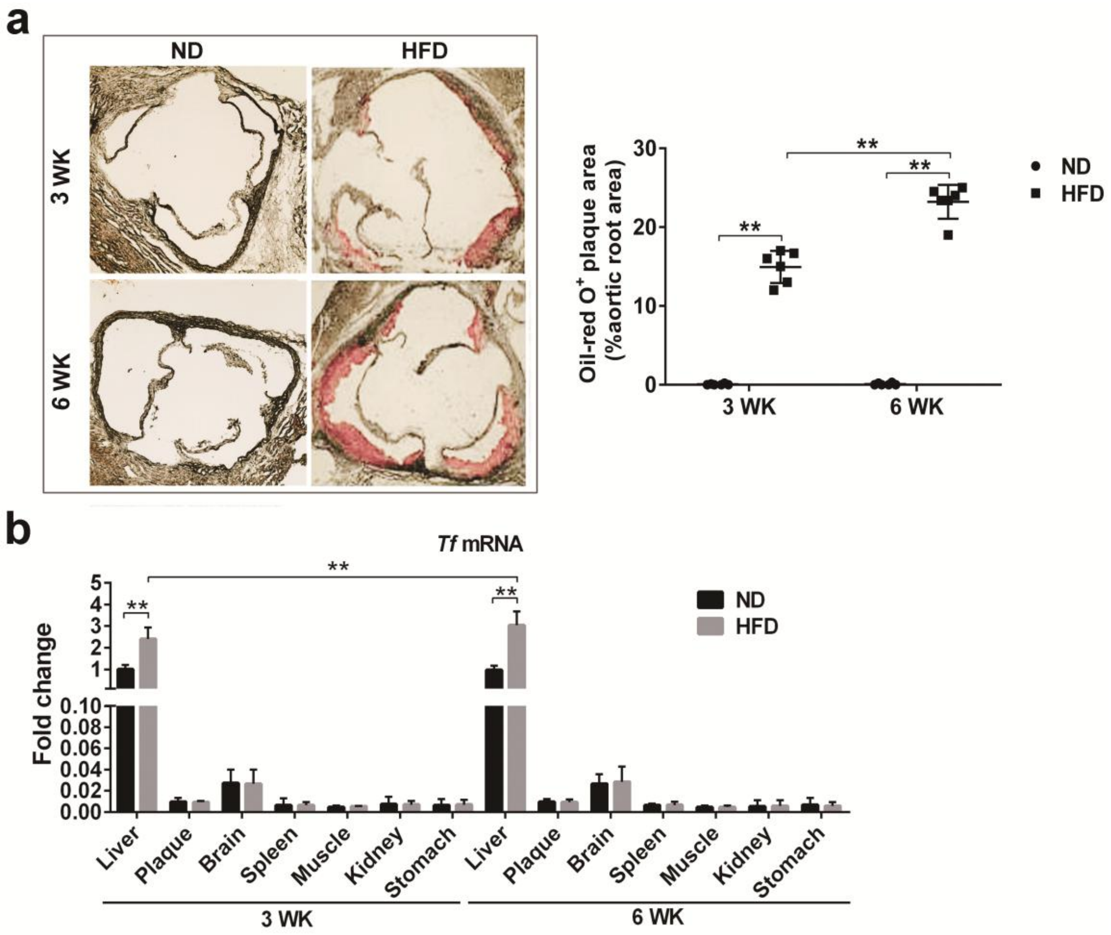

Fig. S3 HFD-induced atherosclerosis and tissue distribution of transferrin mRNA in mice. (a) *Apoe*$^{-/-}$ mice were fed a normal diet (ND) or high fat diet (HFD) for 3 or 6 weeks (WK), with representative images of oil-red O-stained plaques in aortic root area (left) and quantitative results of stained area (right) shown. **(b)** Transferrin mRNA expression in different tissues (liver, plaque, brain, spleen, muscle, kidney, and stomach) was detected by qRT-PCR. Animal experiments were repeated two independent times. Data represent mean ± SD (n = 6), $^{**}p < 0.01$ by unpaired t-test. Tf: transferrin.
