# Supplementary material for: Transferrin plays a central role in coagulation balance by interacting with clotting factors
Source: Cell Res. 2019 Dec 6;30(2):119–32. doi: 10.1038/s41422-019-0260-6 (PMC7015052; doi:10.1038/s41422-019-0260-6)
Supplement: Supplementary file 5 — Supplementary information, Figure S4 [file 41422_2019_260_MOESM5_ESM.pdf]

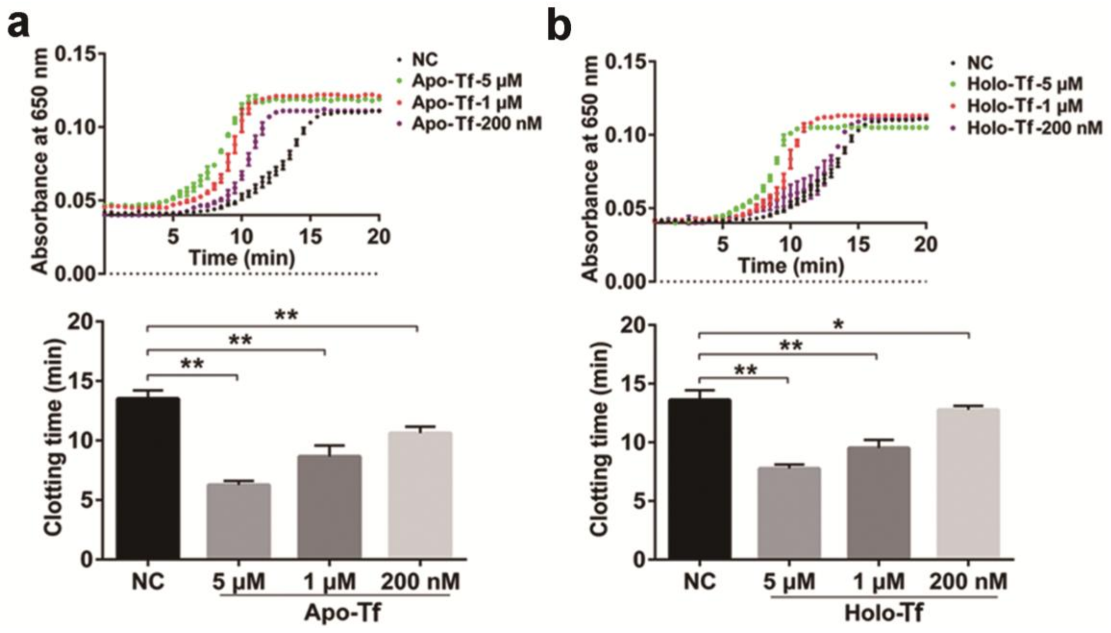

Fig. S4 Effects of apo-transferrin and holo-transferrin on plasma recalcification time. Human plasma recalcification time was shortened by human apo-transferrin **(a)** and holo-transferrin **(b)**. Clotting time (below) was calculated by measuring the time to half maximal increase in absorbance. Data represent mean ± SD of six independent experiments, *$p < 0.05$, **$p < 0.01$ by one-way ANOVA with Dunnett's post hoc test. Tf: transferrin.
