# Supplementary material for: Transferrin plays a central role in coagulation balance by interacting with clotting factors
Source: Cell Res. 2019 Dec 6;30(2):119–32. doi: 10.1038/s41422-019-0260-6 (PMC7015052; doi:10.1038/s41422-019-0260-6)
Supplement: Supplementary file 6 — Supplementary information, Figure S5 [file 41422_2019_260_MOESM6_ESM.pdf]

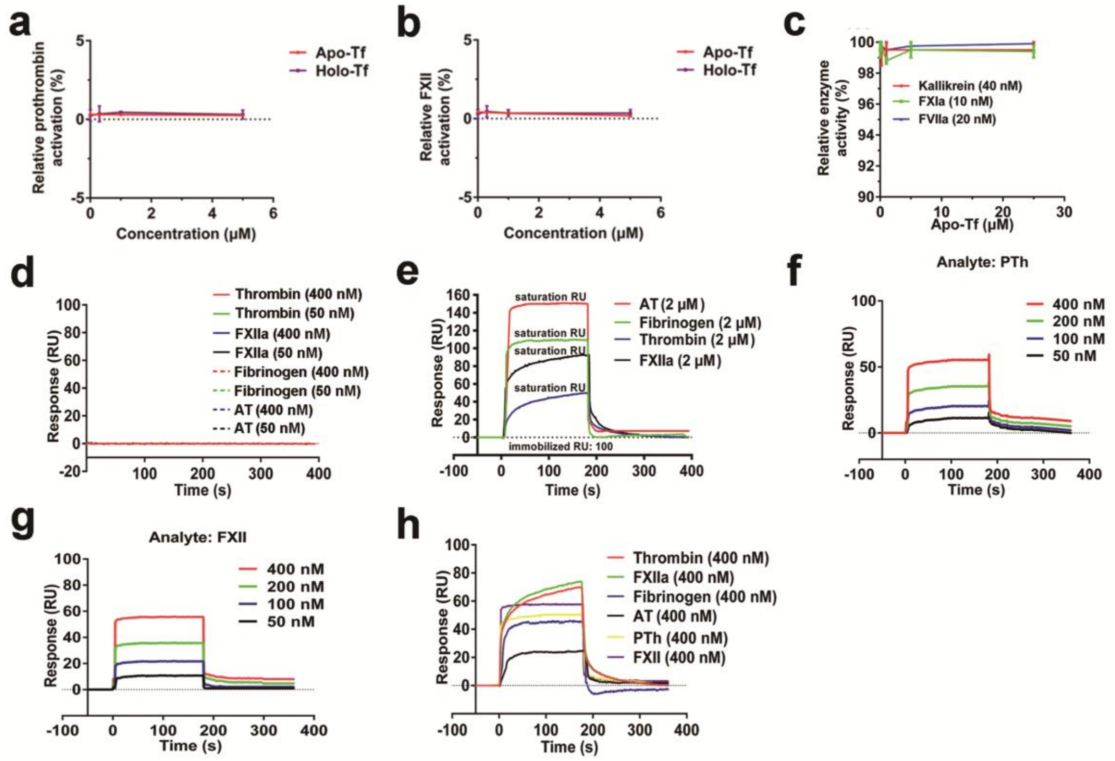

Fig. S5 Effects of apo-transferrin and holo-transferrin on prothrombin/FXII activation and SPR analysis of transferrin and prothrombin/FXII interaction. Both apo-transferrin and holo-transferrin showed no influence on prothrombin **(a)** or FXII **(b)** activation. Data represent mean ± SD of four independent experiments. **(c)** Apo-transferrin showed no influence on kallikrein, FXIa or FVIIa. Data represent mean ± SD of six independent experiments**. (d)** SPR analysis of interactions between human serum albumin (HSA) and thrombin, FXIIa, fibrinogen or antithrombin (AT). **(e)** SPR analysis of binding molar ratio between transferrin and fibrinogen, AT, thrombin, and FXIIa. SPR analysis of interaction between transferrin and prothrombin **(f)** and FXII **(g)**. **(h)** SPR analysis of interactions between holo-transferrin and thrombin, FXIIa, fibrinogen, AT, prothrombin (PTh), and FXII. Tf: transferrin.
