# Supplementary material for: Transferrin plays a central role in coagulation balance by interacting with clotting factors
Source: Cell Res. 2019 Dec 6;30(2):119–32. doi: 10.1038/s41422-019-0260-6 (PMC7015052; doi:10.1038/s41422-019-0260-6)
Supplement: Supplementary file 7 — Supplementary information, Figure S6 [file 41422_2019_260_MOESM7_ESM.pdf]

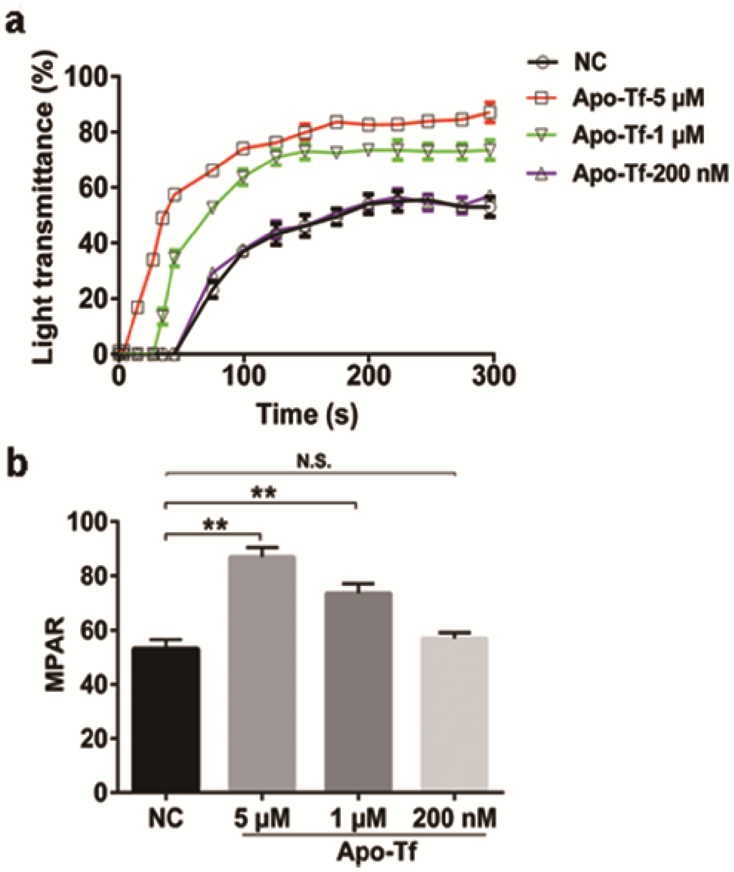

Fig. S6 Effects of apo-transferrin on thrombin-induced platelet aggregation. Transferrin's effect on thrombin-induced platelet aggregation **(a)** and maximum platelet aggregation rate (MPAR) **(b)**. Data represent mean ± SD of six independent experiments, ***p* < 0.01 by one-way ANOVA with Dunnett's post hoc test. Tf: transferrin.
