# Supplementary material for: Transferrin plays a central role in coagulation balance by interacting with clotting factors
Source: Cell Res. 2019 Dec 6;30(2):119–32. doi: 10.1038/s41422-019-0260-6 (PMC7015052; doi:10.1038/s41422-019-0260-6)
Supplement: Supplementary file 8 — Supplementary information, Figure S7 [file 41422_2019_260_MOESM8_ESM.pdf]

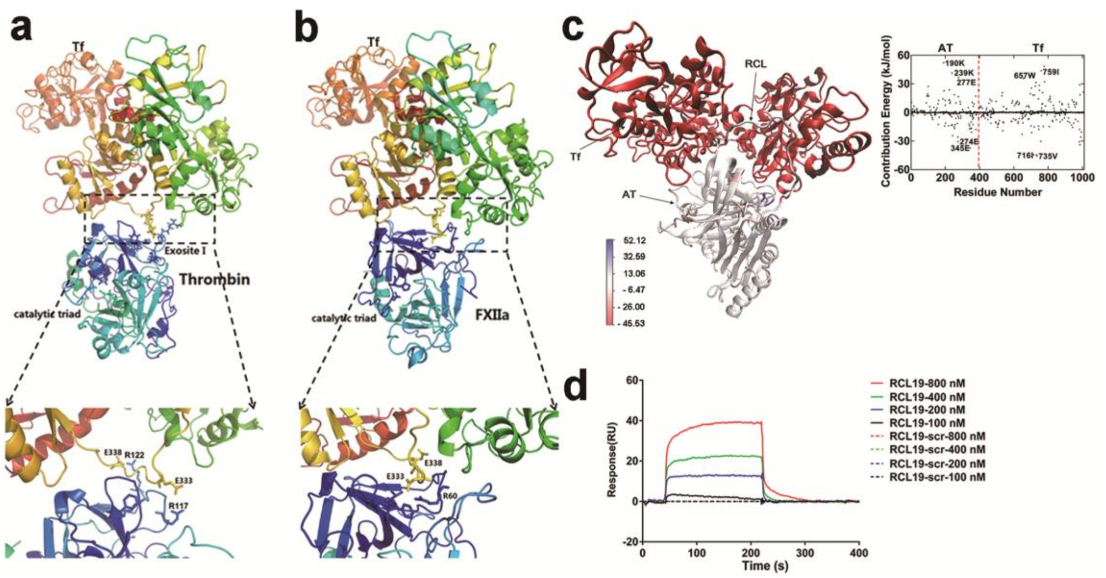

Fig. S7 Interaction of transferrin with thrombin and FXIIa by molecular docking. (a) Transferrin located in the exosite I of human thrombin. Homological model of human thrombin was constructed from known structures (PDB ID: 4NZQ) and indicated by illustration with key residues highlighted by a stick representation. Catalytic triad is marked by colored sticks of side chains. **(b)** Two structures were used to build the working model of human FXIIa (PDB ID 4XE4 and 4XDE). The FXIIa model was aligned along the thrombin model to confirm the possible groove-like binding areas. **(c)** The predicted 3D structure represent the last frames (t = 20 ns) of transferrin (red) and AT (white). The color scale bar represents various energy which marked at residues in AT (left). The plot show the contribution energy of each residues from transferrin-AT complex (right). **(d)** SPR analysis of interaction between transferrin and peptide RCL19 deduced from the sequence of reactive center loop of antithrombin or RCL19-scr (scrambled control of RCL19). Tf: transferrin; AT: antithrombin; RCL: reactive center loop.
