# Supplementary material for: Transferrin plays a central role in coagulation balance by interacting with clotting factors
Source: Cell Res. 2019 Dec 6;30(2):119–32. doi: 10.1038/s41422-019-0260-6 (PMC7015052; doi:10.1038/s41422-019-0260-6)
Supplement: Supplementary file 9 — Supplementary information, Figure S8 [file 41422_2019_260_MOESM9_ESM.pdf]

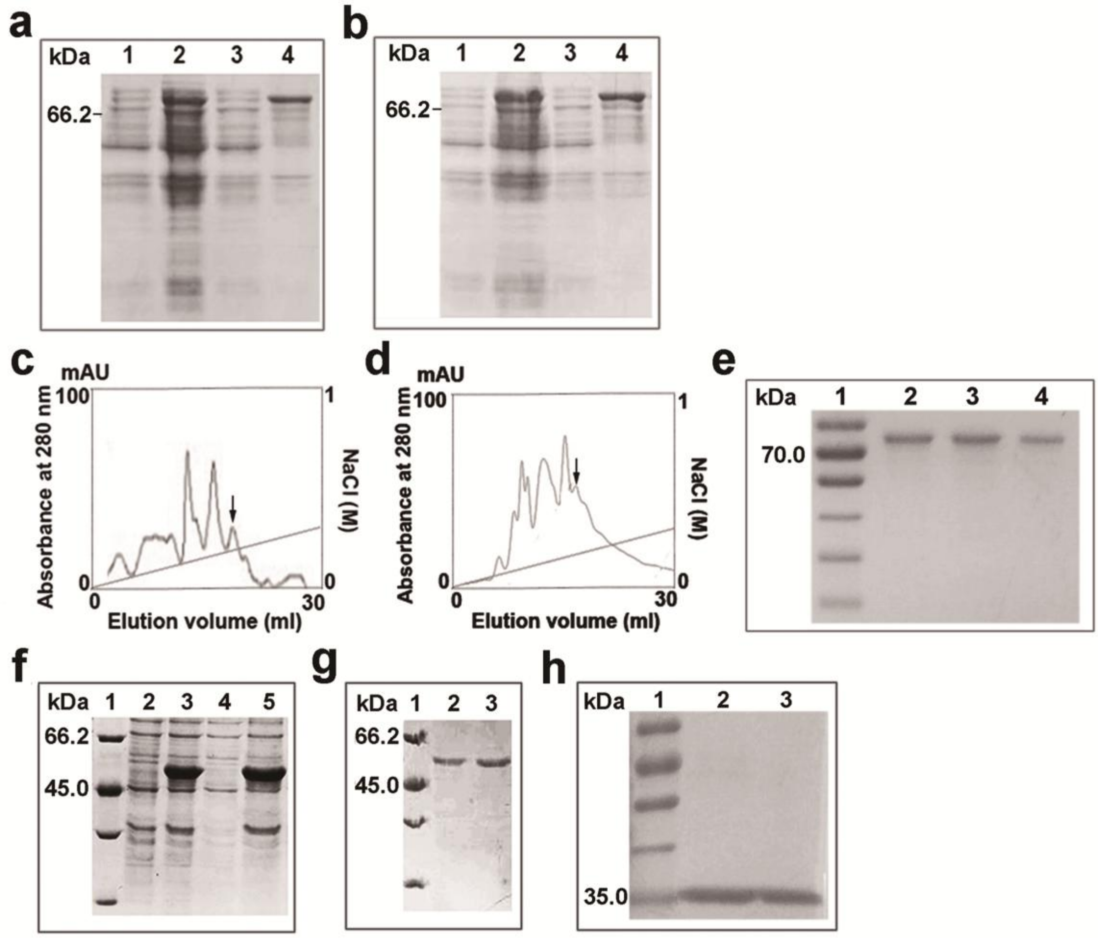

Fig. S8 Recombinant expression and purification of transferrin, thrombin, and their mutants. (a) SDS-PAGE (12%) analysis of production of fused wild-type transferrin after IPTG induction. Lane 1: non-induced; Lane 2: induced; Lane 3: fraction containing wild-type transferrin fusion protein eluted from Ni^{2+} affinity chromatography column cut by enterokinase (5 unit); Lane 4: fraction containing wild-type transferrin fusion protein eluted from Ni^{2+} affinity chromatography column. **(b)** SDS-PAGE (12%) analysis of the production of fused transferrin mutant after IPTG induction. Lane 1: non-induced; Lane 2: induced; Lane 3: fraction containing transferrin mutant fusion protein eluted from Ni^{2+} affinity chromatography column cut by enterokinase (5 unit); Lane 4: fraction containing transferrin mutant fusion protein eluted from Ni^{2+} affinity chromatography column. Fraction containing wild-type

transferrin **(c)** and transferrin mutant **(d)** was eluted by Mono Q column, respectively. Gray line represents line gradient of solvent B (20 mM Tris-HCl, 1 M NaCl, pH 7.8) from 0 to 30% over 30 min. Arrows indicate purified wild-type transferrin and transferrin mutant. **(e)** SDS-PAGE analysis of purified wild-type transferrin and transferrin mutant in 12% gel. Lane 1: protein markers; Lane 2: apo-transferrin (T2036, Sigma); Lane 3: purified wild-type transferrin; Lane 4: purified transferrin mutant. **(f)** SDS-PAGE analysis of production of fused wild-type thrombin and thrombin mutant after IPTG induction. Lane 1: protein marker; Lane 2: wild-type thrombin, non-induced; Lane 3: wild-type thrombin, induced; Lane 4: thrombin mutant, non-induced; Lane 5: thrombin mutant, induced. **(g)** Bound fusion protein of Ni^{2+} affinity chromatography column; Lane1: protein marker; Lane2: fusion protein of wild-type thrombin; Lane3: fusion protein of thrombin mutant. **(h)** Purified wild-type thrombin or thrombin mutant. Lane 1: protein marker; Lane 2: purified wild-type thrombin; Lane 3: purified thrombin mutant.
