# Supplementary material for: Transferrin plays a central role in coagulation balance by interacting with clotting factors
Source: Cell Res. 2019 Dec 6;30(2):119–32. doi: 10.1038/s41422-019-0260-6 (PMC7015052; doi:10.1038/s41422-019-0260-6)
Supplement: Supplementary file 10 — Supplementary information, Figure S9 [file 41422_2019_260_MOESM10_ESM.pdf]

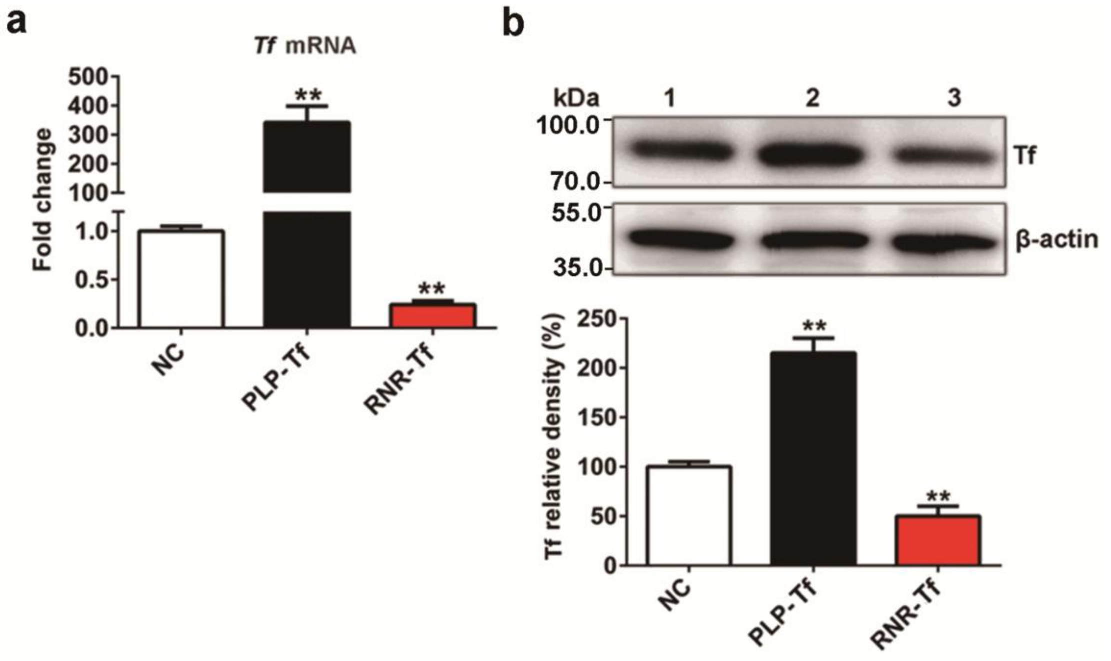

Fig. S9 Construction of transferrin overexpression and knockdown vectors. (a) Transferrin mRNA levels of BNL CL.2 cells after transfection of overexpression or knockdown plasmid of transferrin were determined by qRT-PCR. **(b)** Transferrin levels in BNL CL.2 cells were determined by western blot (top, Lane 1: control (NC), Lane 2: overexpression (PLP-Tf), Lane 3: knockdown (RNR-Tf)). Quantification of the result of western blot is also shown (bottom). Data represent mean ± SD of six independent experiments, ***p* < 0.01 by one-way ANOVA with Dunnett's post hoc test. Tf: transferrin.
