# Supplementary material for: Transferrin plays a central role in coagulation balance by interacting with clotting factors
Source: Cell Res. 2019 Dec 6;30(2):119–32. doi: 10.1038/s41422-019-0260-6 (PMC7015052; doi:10.1038/s41422-019-0260-6)
Supplement: Supplementary file 11 — Supplementary information, Figure S10 [file 41422_2019_260_MOESM11_ESM.pdf]

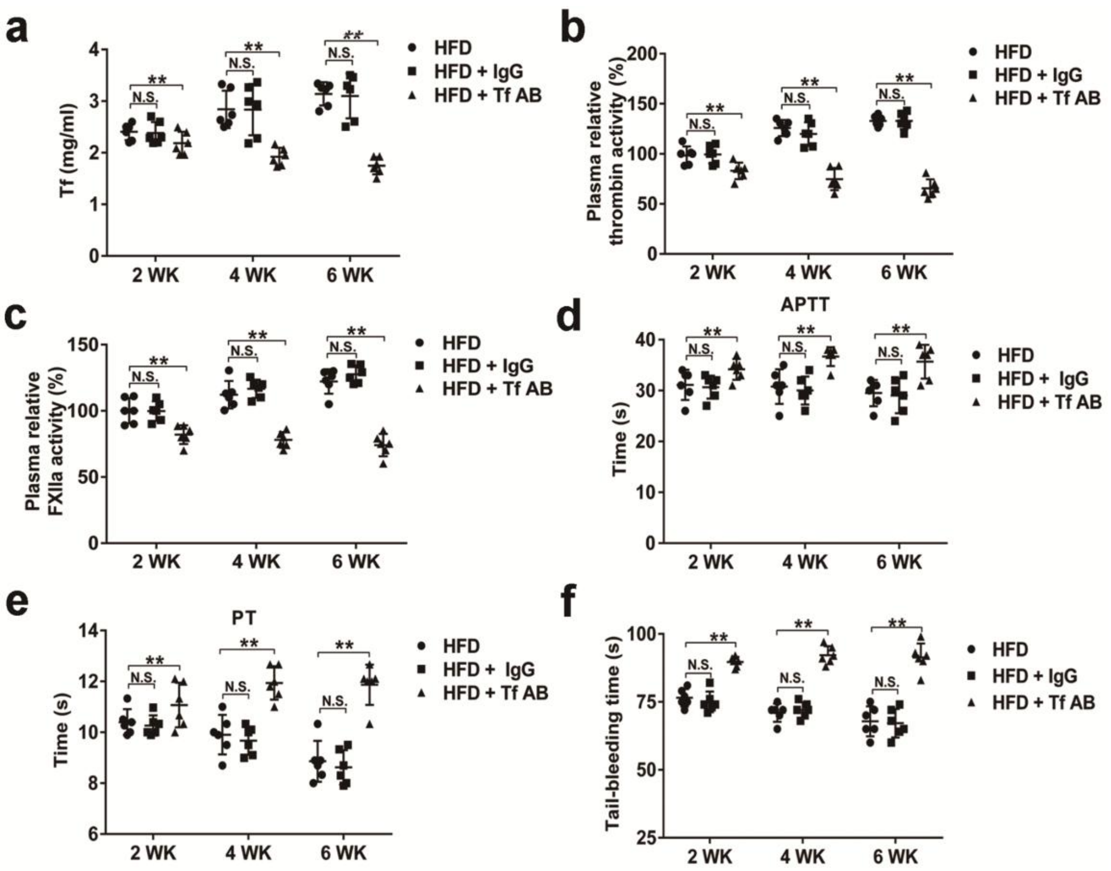

Fig. S10 Anti-transferrin antibody treatment inhibits hypercoagulability *in vivo*. HFD-fed *Apoe*$^{-/-}$ mice were subjected to anti-transferrin antibody (Tf AB) or IgG treatment twice /week for 6 weeks. Plasma concentrations of transferrin **(a)**, relative thrombin **(b),** and FXIIa activities **(c)** in plasma, APTT **(d)**, PT **(e),** and tail-bleeding time **(f)** in *Apoe*$^{-/-}$ mice are shown. Data represent mean ± SD (n = 6), **p < 0.01 by one-way ANOVA with Dunnett's post hoc test. N.S.: no significance; Tf: transferrin.
