# Supplementary material for: Transferrin plays a central role in coagulation balance by interacting with clotting factors
Source: Cell Res. 2019 Dec 6;30(2):119–32. doi: 10.1038/s41422-019-0260-6 (PMC7015052; doi:10.1038/s41422-019-0260-6)
Supplement: Supplementary file 12 — Supplementary information, Table S1 [file 41422_2019_260_MOESM12_ESM.pdf]

Table S1 Clinical features and plasma transferrin concentrations in patients with CHD.

Sex	Age (years)	Main clinical features	Transferrin (mg/ml)
CHD patients			
Female	54	AP, AVLN	5.383
Female	55	AP, AVLN	3.019
Female	61	AP, AVLN	3.770
Male	52	AP, AVLN	6.098
Female	68	AP, AVLN	5.150
Female	71	AP, AVLN	4.244
Male	64	AP, AVLN	2.924
Female	63	AP, AVLN	5.208
Male	43	AP, AVLN	2.949
Male	59	AP, AVLN	7.120
Female	68	AP, AVLN	4.098
Male	74	AP, AVLN	4.600
Male	60	AP, AVLN	3.286
Male	49	AP, AVLN	3.250
Male	57	AP, AVLN	4.186
Male	35	AP, AVLN	3.162
Female	69	AP, AVLN	3.074
Male	70	AP, AVLN	2.920
Female	44	AP, AVLN	3.273
Female	65	AP, AVLN	7.104
Female	79	AP, AVLN	6.300
Male	57	AP, AVLN	3.690
Female	39	AP, AVLN	4.990
Female	71	AP, AVLN	3.180

Female	67	AP, AVLN	3.280
Female	57	AP, AVLN	3.373
Male	61	AP, AVLN	3.373
Female	58	AP, AVLN	5.518
Male	64	AP, AVLN	3.373
Male	70	AP, AVLN	3.295
Male	61	AP, AVLN	3.373
Female	66	AP, AVLN	3.550
Female	71	AP, AVLN	5.050
Female	38	AP, AVLN	3.186
Male	63	AP, AVLN	3.920
Male	29	AP, AVLN	6.560
Female	46	AP, AVLN	5.220
Male	58	AP, AVLN	3.373
Male	39	AP, AVLN	3.200
Male	71	AP, AVLN	5.070
Male	56	AP, AVLN	3.135
Male	67	AP, AVLN	4.940
Female	44	AP, AVLN	3.584
Male	56	AP, AVLN	4.262
Male	41	AP, AVLN	4.286
Female	59	AP, AVLN	3.850
Female	65	AP, AVLN	4.790
Male	78	AP, AVLN	3.399
Male	44	AP, AVLN	6.122
Female	23	AP, AVLN	4.392
Male	73	AP, AVLN	4.103
Male	49	AP, AVLN	7.780
Male	69	AP, AVLN	3.580

Female	63	AP, AVLN	5.379
Female	59	AP, AVLN	3.250
Female	39	AP, AVLN	4.926
Female	59	AP, AVLN	5.670
Male	38	AP, AVLN	3.390
Male	53	AP, AVLN	4.390
Female	67	AP, AVLN	4.350
Female	49	AP, AVLN	3.450
Female	55	AP, AVLN	4.046
Female	73	AP, AVLN	3.201
Male	59	AP, AVLN	4.450
Female	43	AP, AVLN	4.850
Male	69	AP, AVLN	3.562
Female	71	AP, AVLN	2.650
Female	49	AP, AVLN	3.670
Male	45	AP, AVLN	6.114
Male	48	AP, AVLN	3.750
Female	61	AP, AVLN	2.940
Female	65	AP, AVLN	3.342
Male	71	AP, AVLN	5.550
Male	56	AP, AVLN	3.890
Male	65	AP, AVLN	3.506
Male	40	AP, AVLN	3.010
Female	73	AP, AVLN	5.550
Male	39	AP, AVLN	3.950
Female	36	AP, AVLN	2.940
Female	60	AP, AVLN	3.519
Male	34	AP, AVLN	5.500
Female	74	AP, AVLN	5.850

Female	61	AP, AVLN	5.930
Male	59	AP, AVLN	4.400
Female	52	AP, AVLN	3.795
Female	65	AP, AVLN	3.849
Female	55	AP, AVLN	3.620
Male	72	AP, AVLN	6.000
Male	44	AP, AVLN	4.290
Male	64	AP, AVLN	5.500
Female	50	AP, AVLN	3.762
Male	63	AP, AVLN	3.840
Female	59	AP, AVLN	4.400
Female	38	AP, AVLN	3.650
Male	58	AP, AVLN	4.246
Female	51	AP, AVLN	4.849
Male	56	AP, AVLN	3.949
Male	69	AP, AVLN	3.850
Male	59	AP, AVLN	4.600
Female	55	AP, AVLN	3.610
Female	54	AP, AVLN	4.244
Male	70	AP, AVLN	4.430
Male	49	AP, AVLN	4.950
Male	46	AP, AVLN	3.030
Female	67	AP, AVLN	3.800
Female	54	AP, AVLN	3.650
Female	67	AP, AVLN	5.560
Female	52	AP, AVLN	5.510
Male	73	AP, AVLN	5.280
Female	69	AP, AVLN	3.084
Female	28	AP, AVLN	5.926

Male	76	AP, AVLN	4.750
Male	45	AP, AVLN	3.870
Male	50	AP, AVLN	5.379
Female	69	AP, AVLN	4.350
Female	66	AP, AVLN	3.003
Female	67	AP, AVLN	5.379
Male	44	AP, AVLN	5.750
Male	70	AP, AVLN	3.862
Male	59	AP, AVLN	3.820
Controls (healthy volunteers)			
Male	39	NA	2.500
Male	48	NA	2.570
Male	34	NA	3.613
Male	54	NA	2.491
Female	38	NA	2.524
Female	27	NA	3.050
Female	60	NA	2.557
Female	27	NA	2.510
Male	65	NA	2.832
Male	33	NA	2.520
Female	43	NA	2.840
Male	59	NA	3.250
Female	39	NA	2.810
Female	51	NA	3.327
Male	67	NA	3.500
Female	51	NA	3.450
Male	45	NA	3.150
Female	36	NA	3.199
Female	39	NA	3.261

Male	47	NA	2.810
Male	29	NA	2.810
Male	42	NA	3.415
Male	67	NA	2.690
Male	45	NA	3.150
Female	42	NA	2.810
Female	65	NA	2.865
Female	27	NA	2.832
Male	45	NA	2.568
Male	45	NA	2.568
Male	62	NA	3.250
Female	33	NA	3.150
Male	45	NA	2.650
Female	27	NA	2.673
Female	37	NA	2.940
Female	34	NA	2.950
Female	76	NA	2.590
Male	57	NA	2.450
Female	21	NA	2.550
Female	49	NA	2.520
Male	36	NA	2.240
Female	32	NA	2.450
Male	38	NA	2.579
Female	49	NA	2.460
Male	78	NA	2.920
Male	54	NA	2.250
Male	44	NA	2.990
Female	68	NA	2.500
Male	29	NA	3.294

Female	50	NA	2.392
Female	57	NA	2.920
Male	56	NA	2.832
Female	38	NA	3.327
Male	63	NA	2.722
Male	59	NA	2.711
Female	67	NA	2.953
Female	60	NA	2.620
Female	61	NA	2.400
Male	41	NA	2.840
Female	55	NA	2.980
Male	39	NA	2.950
Male	39	NA	2.667
Male	42	NA	3.730
Female	59	NA	2.850
Female	45	NA	2.450
Male	67	NA	3.450
Female	45	NA	2.220
Male	42	NA	2.780
Male	65	NA	2.600
Female	47	NA	2.550
Female	49	NA	2.690
Male	35	NA	2.900
Female	62	NA	2.980
Male	44	NA	2.950
Female	45	NA	2.640
Female	29	NA	3.150
Female	57	NA	2.850
Male	39	NA	2.350

Male	77	NA	2.561
Female	57	NA	2.510
Male	26	NA	2.730
Male	59	NA	2.850
Female	72	NA	2.950
Male	32	NA	2.730
Male	71	NA	2.350
Male	66	NA	3.494
Female	51	NA	2.900
Female	48	NA	3.250
Male	45	NA	3.160
Female	42	NA	2.517
Female	39	NA	3.062
Female	54	NA	3.080
Male	50	NA	3.072
Male	67	NA	3.613
Female	71	NA	3.503
Male	42	NA	3.294
Male	22	NA	2.300
Female	66	NA	2.350
Female	37	NA	2.295
Male	59	NA	2.550
Female	55	NA	2.535
Female	52	NA	3.690
Male	49	NA	2.447
Female	42	NA	2.500
Male	30	NA	3.150
Female	73	NA	2.250
Male	30	NA	2.350

Female	66	NA	2.500
Female	65	NA	2.320
Male	60	NA	2.650
Female	48	NA	2.290
Female	52	NA	2.384
Male	31	NA	2.579
Male	67	NA	3.052
Male	66	NA	2.579
Male	23	NA	2.524
Female	45	NA	2.900
Female	32	NA	3.650
Male	31	NA	2.620
Male	55	NA	3.050
Female	60	NA	2.950

AP: angina pectoris; AVLN: angiographically visible luminal narrowing; NA: not applicable.
