# Supplementary material for: Transferrin plays a central role in coagulation balance by interacting with clotting factors
Source: Cell Res. 2019 Dec 6;30(2):119–32. doi: 10.1038/s41422-019-0260-6 (PMC7015052; doi:10.1038/s41422-019-0260-6)
Supplement: Supplementary file 13 — Supplementary information, Table S2 [file 41422_2019_260_MOESM13_ESM.pdf]

Table S2 Clinical features of atherosclerotic plaque donors.

Sex	Age (years)	Main clinical features
AS plaque donors		
Male	70	AP, AVLN
Male	67	AP, AVLN
Male	65	AP, AVLN
Female	49	AP, AVLN
Female	54	AP, AVLN
Female	60	AP, AVLN
Female	63	AP, AVLN
Male	42	AP, AVLN
Female	50	AP, AVLN
Female	68	AP, AVLN
Male	71	AP, AVLN
Male	63	AP, AVLN

AP: angina pectoris; AVLN: angiographically visible luminal narrowing.
