# Supplementary material for: Transferrin plays a central role in coagulation balance by interacting with clotting factors
Source: Cell Res. 2019 Dec 6;30(2):119–32. doi: 10.1038/s41422-019-0260-6 (PMC7015052; doi:10.1038/s41422-019-0260-6)
Supplement: Supplementary file 14 — Supplementary information, Table S3 [file 41422_2019_260_MOESM14_ESM.pdf]

Table S3 The association rate constant (*Ka*), dissociation rate constant (*Kd*), and equilibrium dissociation constant (*KD*) values for the interaction between apo-transferrin and thrombin, FXIIa, fibrinogen, or antithrombin.

	Ka ($M^{-1}s^{-1}$)	*Kd* (s^{-1})	*KD* (nM)
Tf-thrombin	4.7×10^{5}	3.6×10^{-3}	7.7
Tf-FXIIa	1.8×10^{5}	2.5×10^{-3}	13.9
Tf-fibrinogen	3.4×10^{4}	1.0×10^{-3}	29.0
Tf-AT	2.1×10^{3}	1.1×10^{-3}	524.0

Tf: transferrin; AT: antithrombin.
