# Supplementary material for: Transferrin plays a central role in coagulation balance by interacting with clotting factors
Source: Cell Res. 2019 Dec 6;30(2):119–32. doi: 10.1038/s41422-019-0260-6 (PMC7015052; doi:10.1038/s41422-019-0260-6)
Supplement: Supplementary file 15 — Supplementary information, Table S4 [file 41422_2019_260_MOESM15_ESM.pdf]

Table S4 Iron metabolism indices (iron, ferritin) and erythrocyte indices (MCV, MCH, MCHC) of normal mice (NC), transferrin overexpression (PLP-Tf), knockdown (RNR-Tf), anti-transferrin antibody (Tf AB), and IgG treatment mice.

	Iron (μM/L)	Ferritin (μg/L)	MCV (fL)	MCH (pg)	MCHC (g/L)
NC	16.4 (1.34)	72.2 (2.97)	44.8 (2.12)	14.2 (1.21)	307 (6.73)
PLP-Tf	16.1 (1.96)	71.5 (3.45)	43.5 (3.91)	13.9 (1.98)	310 (5.89)
RNR-Tf	16.5 (1.15)	70.9 (1.95)	44.1 (2.54)	14.4 (1.31)	309 (7.21)
Tf AB	16.0 (1.52)	73 (3.14)	44.0 (2.34)	14.5 (1.34)	312 (6.21)
IgG	16.2 (1.23)	71 (2.34)	45.1 (3.24)	14.1 (2.11)	305 (5.89)

MCV: mean corpuscular volume; MCH: mean corpuscular hemoglobin; MCHC: mean corpuscular hemoglobin concentration. Data represent mean (SD), n = 10.
